# The cognitive structure underlying the organization of observed actions

**DOI:** 10.3758/s13428-022-01894-5

**Published:** 2022-07-05

**Authors:** Zuzanna Kabulska, Angelika Lingnau

**Affiliations:** Department of Psychology, Faculty of Human Sciences, University of Regensburg, Universitätsstraße 31, 93053 Regensburg, Germany

**Keywords:** Action representations, Action categories, Action features, Behavioral similarity

## Abstract

In daily life, we frequently encounter actions performed by other people. Here we aimed to examine the key categories and features underlying the organization of a wide range of actions in three behavioral experiments (*N* = 378 participants). In Experiment 1, we used a multi-arrangement task of 100 different actions. Inverse multidimensional scaling and hierarchical clustering revealed 11 action categories, including *Locomotion*, *Communication*, and *Aggressive actions*. In Experiment 2, we used a feature-listing paradigm to obtain a wide range of action features that were subsequently reduced to 59 key features and used in a rating study (Experiment 3). A direct comparison of the feature ratings obtained in Experiment 3 between actions belonging to the categories identified in Experiment 1 revealed a number of features that appear to be critical for the distinction between these categories, e.g., the features *Harm* and *Noise* for the category *Aggressive actions*, and the features *Targeting a person* and *Contact with others* for the category *Interaction*. Finally, we found that a part of the category-based organization is explained by a combination of weighted features, whereas a significant proportion of variability remained unexplained, suggesting that there are additional sources of information that contribute to the categorization of observed actions. The characterization of action categories and their associated features serves as an important extension of previous studies examining the cognitive structure of actions. Moreover, our results may serve as the basis for future behavioral, neuroimaging and computational modeling studies.

**Supplementary Information:**

The online version contains supplementary material available at 10.3758/s13428-022-01894-5.

## Introduction

Being able to tell whether we are greeted or attacked by another person is a crucial skill for our survival. What are the key categories underlying the organization of observed actions, and what kind of information do we exploit to quickly categorize and understand actions performed by other people? There is a long tradition in asking this question in the domain of object categories. Aristotle (Aristotle, 1995/350 BCE) argued that categories can be distinguished on the basis of the presence or absence of relevant features (such as a tail or a wing). More recent views emphasize the similarity of weighted features (e.g., Cree & McRae, 2003; Vinson et al., 2003). Some authors pointed out the importance of sensory, functional, motor, and manipulation features (e.g., Binder et al., 2016; Cree & McRae, 2003; McRae et al., 2005; Vigliocco et al., 2004; Vinson & Vigliocco, 2008). According to this view, a cat will be distinguished from other animals by visual features, such as its posture and whiskers, whereas a chair will be distinguished from other non-living objects by functional features, such as that it is something to sit on. Binder et al. (2016) emphasized the role of features with known corresponding neural representations, such as sensory, spatial, and temporal features. In contrast to views that emphasize the role of different *types* of features, some authors argued that categories differ with respect to the *distribution* and *correlation of features* across different categories (for review, see Mahon & Caramazza, 2009).

A vast number of neuroimaging studies have reported a preference for object categories such as faces, houses, tools, and animals in ventral stream regions (Downing et al., 2001; Kanwisher et al., 1997; Malach et al., 1995). These results are supported by corresponding category-selective deficits in patients (e.g., Humphreys & Rumiati, 1998; Moscovitch et al., 1997). It has been argued that category selectivity reported in the ventral stream is at least partially due to visual features that systematically differ between object categories (for review, see e.g., Bracci et al., 2017). In line with this view, it has been proposed that object categories are represented by distributed feature maps rather than by functionally specific regions (see e.g., Haxby et al., 2001). Recent neuroimaging studies that directly compared the organization according to features and object categories revealed that features alone are not sufficient to account for the category-based structure (e.g., Jozwik et al., 2016).

To which degree do the principles regarding the cognitive organization of objects according to features and categories described above apply to the organization of observed actions? Vigliocco et al. (2004) reported that both for object and action words, feature-based similarities can predict human similarity judgments. However, as pointed out by Vinson and Vigliocco (2008), objects and actions differ with respect to a number of aspects. Importantly, objects typically can be understood in isolation and often can be identified on the basis of a small number of features that show a strong correspondence with specific categories (e.g., beak and wings would be typical features of a bird). By contrast, many actions can only be understood on the basis of their relation towards objects (e.g., opening a door) or other agents (e.g., hugging someone), and they can be performed in various different ways (e.g., eating with a fork, with chop sticks, or with both hands). Moreover, actions differ with respect to the desired goal state, which can be described along a concrete-abstract continuum, from a change of posture (e.g., getting up) or location (e.g., riding the bike) towards a change of an object configuration (e.g., opening a book) to a change of a mental state (e.g., listening to music; see also Hamilton & Grafton, 2006; Vallacher & Wegner, 1985; Wurm & Lingnau, 2015). Consequently, determining the principles underlying the organization of actions is challenging and has only recently started to attract a growing level of attention in the literature. As an example, Watson and Buxbaum (2014) revealed two dimensions that underlie the organization of actions involving tools, namely, the amount of arm movement and the hand posture. Using standard univariate analyses of fMRI data, different types of information pertaining to observed actions have been reported to engage different brain regions, such as the ventral premotor cortex in response to the effector type used in an action (e.g., foot, hand, mouth) and the parietal cortex in response to movement direction (Jastorff et al., 2010) and different types of actions (Abdollahi et al., 2013; Ferri et al., 2015). Using multivariate pattern analysis of fMRI data, Wurm et al. (2017) reported a distinction between person-directed and object-directed actions in the lateral occipito-temporal cortex (LOTC). Using a multi-arrangement task, Tucciarelli et al. (2019) identified a number of categories according to which observed actions are organized behaviorally, including locomotion, communicative actions, food-related actions, and cleaning-related actions. Using multiple-regression representational similarity analysis (RSA) of fMRI data, the authors identified a region in the LOTC that reflected this category-based similarity structure while accounting for a number of other components such as the context, body parts, kinematics, and low-level visual features. Using a similar approach, Tarhan et al. (2021) revealed an action processing hierarchy in the visual system and emphasized the importance of actors’ goals. Finally, Tarhan and Konkle (2020) revealed brain networks recruited during the processing of videos of actions that carry information regarding body parts and the target of an action.

Understanding how objects and actions are perceived and which features are useful in this process is important also beyond the field of cognitive neuroscience. Advances in the knowledge about the anatomy and function of the visual system proved to be useful in computer science (Cichy et al., 2019; Hassabis et al., 2017; Wardle & Baker, 2020), first allowing to create a mathematical model of an artificial neuron (McCulloch & Pitts, 1943) that later became a building block for a single-layer perceptron (Rosenblatt, 1958) and for currently widely used deep neural networks (DNNs) (Cios, 2018; Rumelhart et al., 1986). Likewise, methodological developments in the field of human object recognition contributed to creating better computational models. For instance, DNNs differ from humans in the source of information used for object classification, relying more on the texture of images rather than on the shape. It has been shown that teaching a DNN to classify objects based on shape improved the network’s performance, resulting in a more accurate imitation of human-like judgments (Geirhos et al., 2019). Thus, understanding which features are important for humans to distinguish between objects and actions and to categorize them is crucial for building artificial models that can mimic cognitive processes.

In sum, whereas previous studies revealed a number of potential organizing principles of observed actions, we are lacking a thorough investigation of the categories and features that are used to identify and distinguish between them. The current study aims to address this gap in the literature. In Experiment 1, we used a multi-arrangement task of 100 actions depicted as static images in combination with inverse multidimensional scaling analysis (Kriegeskorte & Mur, 2012) to obtain the category-based structure that captures similarities between different actions. In short, participants were asked to arrange a set of action images on a computer screen in a way that the distances between the images reflect action similarity. In Experiment 2, we performed a free feature-listing experiment for the same actions as in Experiment 1 (using verbal material) that resulted in a wide range (approx. 6000) of action features. Subsequently, we reduced that list to 59 key features, for which we collected ratings in Experiment 3. By combining these ratings with the results of Experiment 1 we reveal critical features that contribute towards the distinction between action categories, and we show how features may contribute to the category-based organization of observed actions.

## Experiment 1

The aim of Experiment 1 was to determine the categories underlying the organization of observed actions.

### Methods

#### Participants

Twenty participants took part in the experiment (ten females; mean age = 22 years, age range = 19–27). Participants were financially reimbursed for their participation. Experimental procedures were approved by the ethics committee at the University of Regensburg.

#### Materials

We used 100 images of a wide range of daily actions. The initial list of actions was chosen from a study of Vinson and Vigliocco (2008), which reported semantic feature production norms for a wide range of verbs (*N* = 216) referring to events from different semantic fields such as manner of motion, body motion, and communication. We selected verbs that present typical, well-known daily actions that are easy to depict as static images, e.g., *brushing hair*, *driving a car*, *eating*. Details regarding the selection of action word and images and the full list of actions are provided in the Supplementary Materials (Sections S.1.1, S.1.2, and Table S1).

#### Procedure

The multi-arrangement experiment (Kriegeskorte & Mur, 2012) was conducted at the University of Regensburg. Participants were asked to position images on the screen in such a way that the distance between the images reflected their perceived similarity in terms of their meaning, rather than the background (e.g., an outdoor scene or a kitchen) or the overall composition of the picture (see also Tucciarelli et al., 2019). As an example, actions with a very similar meaning (e.g., *running* and *walking*) should be positioned close to each other, while actions with very different meanings (e.g., *running* and *taking a shower*) should be positioned further apart (see Fig. S2 for an illustration). In the first trial, all the 100 action images appeared on a so-called circular “arena”. The arrangement was performed by drag-and-drop using the mouse and, when all the images were sorted inside the arena, the participant was asked to press a button, which started the next trial. In each trial, the program determined the dissimilarities between all the actions in Euclidean space on the basis of their pairwise distances on the screen. The program updates the estimates of the pairwise distances, such that the pairwise dissimilarity evidence increases progressively. In subsequent trials, images were sampled from the original stimulus set by picking those with the least amount of evidence. A detailed description of the multi-arrangement procedure can be found in Kriegeskorte and Mur (2012). The average duration of the experiment was 120 min.

#### Data analysis

Data analysis was carried out in MATLAB (The MathWorks Inc., Natick, MA, USA). Separately for each participant, Euclidean distances for all 4950 pairwise comparisons of the 100 actions were reshaped into a vector, and, subsequently, averaged across participants. The obtained vector was transformed into a 100 x 100 representational dissimilarity matrix (RDM; see Fig. S3), depicting the relation between actions for all possible pairs of actions. To visualize the results in 2D, we used non-metric multidimensional scaling (MDS; criterion: metric stress, stress value = 0.237, distance measure: Euclidean).

To access the structure underlying the representation of observed actions, we conducted hierarchical clustering analysis (see also Tucciarelli et al. (2019) for a similar approach). First, to reveal the metric that is best suited for clustering the data, we calculated the cophenetic correlation coefficients (Sokal & Rohlf, 1962; function *cophenet* in MATLAB) for different metrics. This method allows computing the correlation between cluster distances (so-called cophenetic distances generated by the *linkage* function in MATLAB) and actual Euclidean distances between the clusters (generated by the *pdist* function), enabling to assess whether the chosen clustering method reflects the original distances accurately. We obtained the highest value (cophenetic correlation = 0.854) for the *average* method (unweighted pair group method with arithmetic mean (UPGMA), Sokal & Michener, 1958). The resulting method indicates which algorithm will be used to group the data points into clusters and compute between-cluster distances (*linkage* function). UPGMA is an agglomerative method for hierarchical clustering that starts with each data point being its own single cluster and, moving bottom-up, forms a cluster from two clusters for which the average distance is the smallest. The average distance is calculated as the mean distance between all the members of each group. Second, to determine the number of clusters which best describe the dataset, we computed the silhouette index (*si*) (Rousseeuw, 1987) in a range from 3 to 50 (which corresponds to half of the number of stimuli) (Fig. S4). In brief, the silhouette index reveals how appropriate a clustering solution is, by taking one data point at a time and comparing its distances with all other data points within the cluster to the between-cluster distances of the nearest cluster. The silhouette index ranges from – 1 to 1, where 1 indicates the best clustering of the data, whereas 0 represents a random clustering.

To obtain labels for the action categories revealed by hierarchical clustering, we conducted an online experiment in a separate group of participants. We only used clusters that contained at least two actions (which was the case for 11 out of 12 clusters). Participants were asked to provide category labels based on actions belonging to each category. We selected the final category labels on the basis of their frequency (see Section S.1.5 in the Supplementary Materials for a detailed description of the Category naming experiment).

### Results

**Fig. 1:**
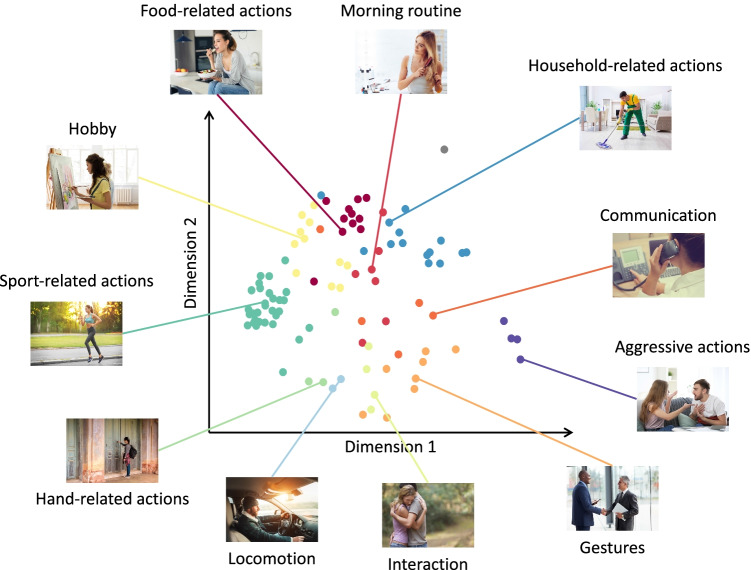
Action categories revealed by the multi-arrangement experiment and their arrangement in a two-dimensional space. Each *dot* represents one action, *colors* correspond to different action categories. For each category, a representative action with the corresponding category label is shown for ease of visualization. The *gray dot* indicates the action *smoking* belonging to the single-action cluster that was not considered in the subsequent experiment (see Section *Experiment *1*, Data analysis*)

Participants sorted actions according to several clusters; according to the silhouette index, the optimal number of clusters was 12 (*si* = 0.23; see Fig. S4). As mentioned in Section *Experiment *1*, Data analysis*, we removed one cluster since it only consisted of one single action. The remaining 11 action categories were labeled as follows: *Aggressive actions*, *Communication*, *Food-related actions*, *Gestures*, *Hand-related actions*, *Hobby*, *Household-related actions*, *Interaction*, *Locomotion*, *Morning routine*, and *Sport-related actions*. Information about the categories and the corresponding actions is provided in Table S3. Figure 1 presents a 2D MDS solution depicting the 11 action categories together with the corresponding labels. Clusters along the first dimension appear to be organized into pleasant (*Sport-related actions*, *Hobby*) and non-pleasant actions (*Household-related actions*, *Aggressive actions*). The second dimension might represent the presence (*Food-related actions*, *Morning routine*, *Household-related actions*) or absence (*Hand-related actions*, *Locomotion*, *Interaction*, *Gestures*, *Aggressive actions*) of a tool.

### Discussion

Experiment 1 revealed 11 action categories, namely *Aggressive actions*, *Communication*, *Food-related actions*, *Gestures*, *Hand-related actions*, *Hobby*, *Household-related actions*, *Interaction*, *Locomotion*, *Morning routine*, and *Sport-related actions*. The obtained categories provide an extension of the five categories obtained from 28 daily life activities by Tucciarelli et al. (2019). We discuss these results in relation to the existing literature in the General discussion.

## Experiment 2

In Experiment 2, we aimed to explore the feature-based organization of actions. It is not obvious which features should be used to best describe actions, and to distinguish them from other actions (see also Hebart et al., 2020; Zheng et al., 2019). One possibility would be to select a number of features that are either theoretically motivated, or that have been proposed in previous studies (e.g., Binder et al., 2016; Orlov et al., 2014; Tarhan & Konkle, 2020; Vigliocco et al., 2004; Vinson & Vigliocco, 2008; Watson & Buxbaum, 2014; Wurm et al., 2017; Yang et al., 2017). We reasoned that a disadvantage of such an approach is the risk to miss relevant features. To minimize this risk, we decided not to base the selection of features on the basis of previous studies alone, but to support this step with an exploratory feature generation task. To this aim, we asked a separate group of participants to provide action features for each of the 100 actions used in Experiment 1 using a free feature-listing paradigm. Subsequently, we used the obtained features in combination with features proposed in previous studies to select a subset of features to be used in an explicit feature rating of actions (Experiment 3).

### Methods

#### Participants

Forty participants (15 females; mean age = 23 years, age range = 18–36 years), recruited among students at the University of Regensburg, took part in the study and were financially reimbursed for their time. Experimental procedures were approved by the ethics committee at the University of Regensburg.

#### Apparatus

We collected action features for 100 actions using an online survey (https://www.soscisurvey.de/).

#### Materials

Stimuli consisted of the same 100 actions as used in Experiment 1, depicted as German verbs in their infinitival form (see Table S1).

#### Procedure

##### Free feature-listing experiment

In each trial, participants were provided with an action word (e.g., “laufen – *to run*”) and were asked to generate features that are typical for that action, which are relevant to understand it, and by which the action can be distinguished from others. Participants were instructed to provide at least five features per action, and they were provided with example features for two actions that were not part of the experiment (see Supplementary Materials, Section S.2.1, for the full instruction). Each participant was asked to provide features for 25 actions, such that we obtained features from ten participants for each of the 100 actions. The duration of the experiment was approximately 25 min.

##### Selection of themes and key features

The obtained list (*N* = 5683 features) consisted of duplicate features as well as features that were phrased differently but carried the same or a very similar meaning (e.g., “Werkzeug – *tool*” and “Werkzeuge – *tools*”). Whereas the identification of distinct features specific for each action or action category can without doubt be useful as well (e.g., Zhuang & Lingnau, 2021), the focus of the current study was to identify more general features that are suitable for the collection of ratings across a wide range of actions. This was the reason to reduce the obtained set of features. Reduction of features was performed in several steps. First, separately for each of the 100 actions, a native German speaker collapsed duplicates of features or features with similar meaning (e.g., singular or plural nouns), while keeping different grammatical forms (e.g., “Anspannen – *to tighten*” and “Anspannung – *tension*”) separate. This resulted in *N* = 4504 features; the corresponding list is provided on osf [https://osf.io/73v58/], table ‘Action features – 100 actions’. Next, we collapsed duplicates of features across the whole dataset, which resulted in 3243 unique features (see table ‘Action features – unique’ on osf). This set of unique features consisted of single words and phrases that differed in terms of their grammatical class (e.g., nouns, verbs, adjectives), and the level of abstraction (ranging from concrete, specific features such as *lifting an arm* to abstract features, such as *communication*). Given this large variety and number of features, our next goal was to reduce the number of collected features to the most crucial ones, while at the same time keeping as much information from the collected dataset as possible. To this aim, we grouped the features into “themes” that keep conceptually related features together (see Table 1 and Fig. S6). To identify themes according to which these features can be organized, we conducted an exploratory analysis of the dataset. To do so, the same native German speaker and one of the authors thoroughly went through the data set and, to avoid subjectivity, independently came up with main themes that could best describe the content of the dataset. In the next step, following previous studies, we selected themes that could be backed-up by the features provided by the participants in Experiment 2. We chose the following themes: *Body parts* (Orlov et al., 2014; Tarhan & Konkle, 2020; Yang et al., 2017), *Object-directedness* (Tarhan & Konkle, 2020; Wurm et al., 2017; Yang et al., 2017), *Type of limb movement* (Tranel et al., 2003; Watson & Buxbaum, 2014), *Posture* (Peelen & Downing, 2007), *Location* (Wurm & Schubotz, 2017), *Keeping balance* (Vaessen et al., 2018), *Harm* (Tranel et al., 2003; Binder et al., 2016), *Change of location* (Vinson & Vigliocco, 2008), *Duration* (Tranel et al., 2003; Binder et al., 2016; Yang et al., 2017), *Contact with others* (Vinson & Vigliocco, 2008; Binder et al., 2016), *Use of force* (Watson & Buxbaum, 2014; Yang et al., 2017), *Goal-directedness* (Hamilton & Grafton, 2007; Wurm et al., 2016), *Pace and Concentration* (Binder et al., 2016), *Noise* (Tranel et al., 2003; Vinson & Vigliocco, 2008), and *Valence* (Tranel et al., 2003; Binder et al., 2016). The remaining themes (i.e., *Trajectory*, *Water*, and *Season-dependence*) were selected based on the collected features. We decided to include these themes because they were mentioned frequently.

**Table 1:**
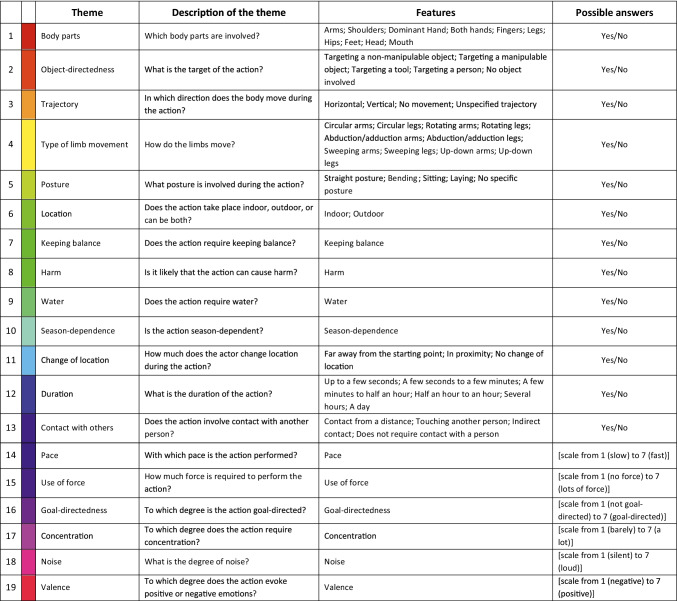
List of action features grouped by the corresponding themes

Themes and key features selected based on the features obtained from the free feature-listing experiment (see Section *Experiment* *2**, Procedure, Selection of themes and key features* for a detailed description of the procedure) and based on the existing literature. The selection resulted in 59 key features grouped into 19 themes. Each of the themes is marked by a different color**.** Thirteen out of the 19 themes consist of a set of binary features (possible answers: “Yes/ No”). The other six themes (e.g., *Pace*, *Use of force*) correspond to features that can be judged on a continuous scale (e.g., *Pace*, from 1 = slow to 7 = fast). Nine themes contain multiple features whereas the remaining ten themes consist of one single feature only. In the latter case, we refer to theme and feature with the same name (e.g., *Pace*). Questions describing each theme are provided to better illustrate the meaning of the theme. The last column contains possible answers (see *Experiment* *3* for details)

In the next step, for each theme, we identified features belonging to each theme and selected those that were frequently mentioned by the participants. Based on these features, we came up with key features. This way, we aimed to preserve detailed features from participants’ responses while keeping them organized within groups containing conceptually related information.

Because of the wide range of features obtained from the feature-listing experiment, we decided on two different types of key features: binary features that are reasonable to be judged with a yes/ no answer (e.g., *Arms*) and continuous features for which we considered it more useful to ask for a rating on a scale (e.g., *Pace*) (see Table 1 for a better understanding of the key features and the possible answers).

### Results

From the free feature-listing experiment, we obtained 5683 features describing 100 daily actions. Due to the nature of the task, the features varied in a number of different ways, e.g., in terms of the grammatical class (verbs, nouns, adjectives), the phrase length (single words, phrases, sentences), and the level of abstraction. The complete list of features separately for each of the 100 actions as well as a table with all the unique features across the 100 actions are available at https://osf.io/73v58.

In the second part of Experiment 2, we selected 59 key features (such as *Arms*, *Hands*, *Targeting a tool*, *Targeting a person*), which we divided into 19 broader themes: *Body parts*, *Object-directedness*, *Trajectory*, *Type of limb movement*, *Posture*, *Location*, *Keeping balance*, *Harm*, *Water*, *Season-dependence*, *Change of location*, *Duration*, *Contact with others*, *Pace*, *Use of force*, *Goal-directedness*, *Concentration*, *Noise*, and *Valence*. Thirteen of the themes contained binary features, i.e., those that can be either involved in an action or not (e.g., *Arms*, *Legs*), whereas the remaining themes contained features that could be described on a continuous scale (e.g., *Pace*, from slow – 1 – to fast – 7 –). Moreover, some of the themes contained multiple features (e.g., *Arms*, *Shoulders*, *Legs* etc. for the theme *Body parts*) whereas some contained one feature only (e.g., *Keeping balance*). For the latter, we refer to the themes and the features with the same name. The full list of themes and their corresponding features is provided in Table 1.

### Discussion

In Experiment 2, we obtained a wide range of different features (5683 in total) that human participants associate with different actions. We subsequently summarized that list to 59 features, organized by different themes, to be used in an explicit feature rating (Experiment 3). The reduced set of features covers varying levels of abstractness, ranging from concrete features, e.g., *Body parts*, *Type of limb movement*, *Posture* to more abstract features such as *Harm*, *Valence* and *Goal-directedness* (Wurm et al., 2016). Moreover, the features cover different semantic domains (see e.g., Binder et al., 2016). Specifically, features are part of the sensory (e.g., *Noise*), motor (e.g., *Type of limb movement*), space (e.g., *Location*, *Change of location*), time (*Duration*), social (*Contact with others*), emotion (*Valence*), and drive (*Goal-directedness*) domains. Both the complete set of features provided by the participants as well as the selected set of features may serve as a starting point for future studies concerned with the behavioral and neural correlates of specific features, and for computational models aimed at the recognition of human actions.

## Experiment 3

The goal of Experiment 3 was (1) to identify critical features that are used to distinguish between different action categories, and (2) to directly compare the feature- and category-based organization of actions. To this aim, we collected ratings for 59 action-related features obtained from Experiment 2.

### Methods

#### Participants

A total of 273 participants took part in the rating experiment (231 females; mean age = 28 years, age range = 16–67 years) and were financially reimbursed for their time. Experimental procedures were approved by the ethics committee at the University of Regensburg.

#### Apparatus

The study was conducted as an online survey (www.soscisurvey.de).

#### Materials

Stimuli consisted of the same 100 actions as used in Experiments 1 and 2, depicted as German verbs in their infinitival form (see Table S1 for a list of all actions). The actions were rated based on 59 key features selected in Experiment 2.

#### Procedure

Participants were asked to provide ratings for 59 features (see Table 1). Instructions provided to the raters can be found in Section S.3.1. For the features of 13 of the themes, binary answers (“Yes” or “No”) were required, whereas features for the remaining six themes had to be judged on a scale from 1 to 7 (e.g., 1: ”Not at all”, 7: “Very much”) (see Table 1 for details). In addition to the instruction, participants were provided with an example action (not used in the study) with corresponding example ratings.

For the features from the themes *Body parts*, *Object-directedness*, *Trajectory*, *Type of limb movement*, *Posture*, *Location*, *Keeping balance*, *Duration*, *Contact with others*, *Pace*, *Use of force*, and *Goal-directedness* 17 participants rated 25 action words each (425 ratings in total), which took approximately 45 min. To reduce the amount of time per participant, another set of 107 participants rated five action words each (535 ratings in total), which took about 10 min. For features from the remaining themes, we collected ratings from a separate group of 149 participants. Each participant rated five action words (745 ratings in total) and the full experimental session lasted approximately 10 min. The set of actions chosen for each participant was randomized.

#### Data analysis

All subsequent analyses, unless stated otherwise, were conducted using MATLAB (The MathWorks Inc., Natick, MA, USA). We obtained 1680 ratings in total, with the number of ratings per action ranging between seven and eleven. First, we reduced the redundancy within the features (see Supplementary Materials, Section S.3.2.1, for details). Since ratings differed depending on the theme (either “Yes/No” answers or ratings on a scale), we transformed “Yes/No”-answers to values of 1 or 0 and rescaled values of continuous ratings to a range from 0 to 1. To avoid multicollinearity, we removed features that were highly correlated (see Section S.3.2.2). The final set comprises of 44 features.

##### Multi-feature model

**Fig. 2:**
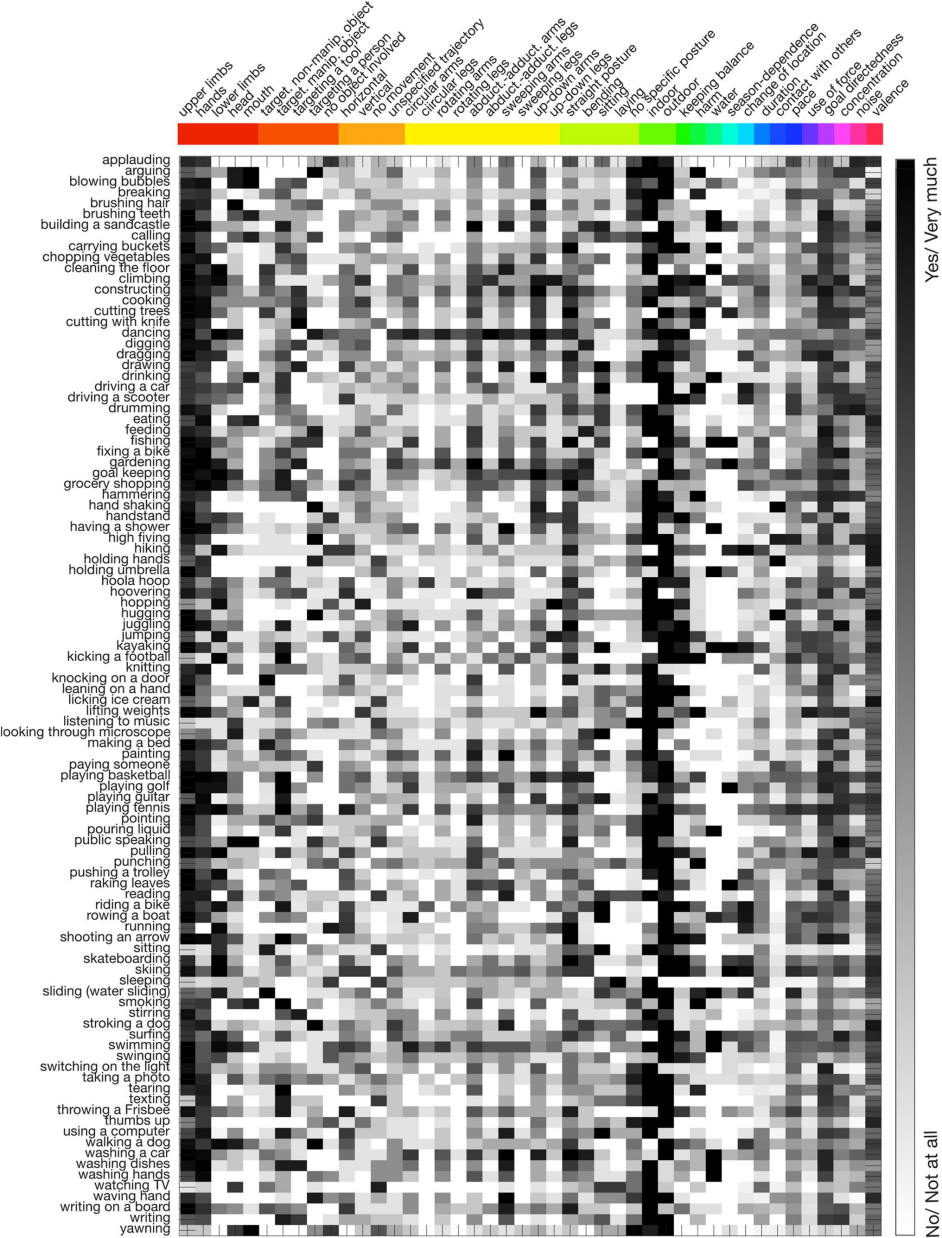
Multi-feature model. The model depicts ratings for 100 actions (*rows*) based on 44 features (*columns*). Different shades of gray indicate the mean rating of a feature (*black*: high rating, *white*: low rating). Features belonging to the same theme are depicted by the same color on the top of the figure (same color code as Table 1)

To depict which features are important for different actions, we averaged ratings across participants and created a multi-feature model (Fig. 2). Additionally, we selected four exemplary features and showed actions that received minimum and maximum ratings (Fig. S9) for an additional visualization of the results of the feature rating.

##### Feature-based representations of action categories

**Fig. 3:**
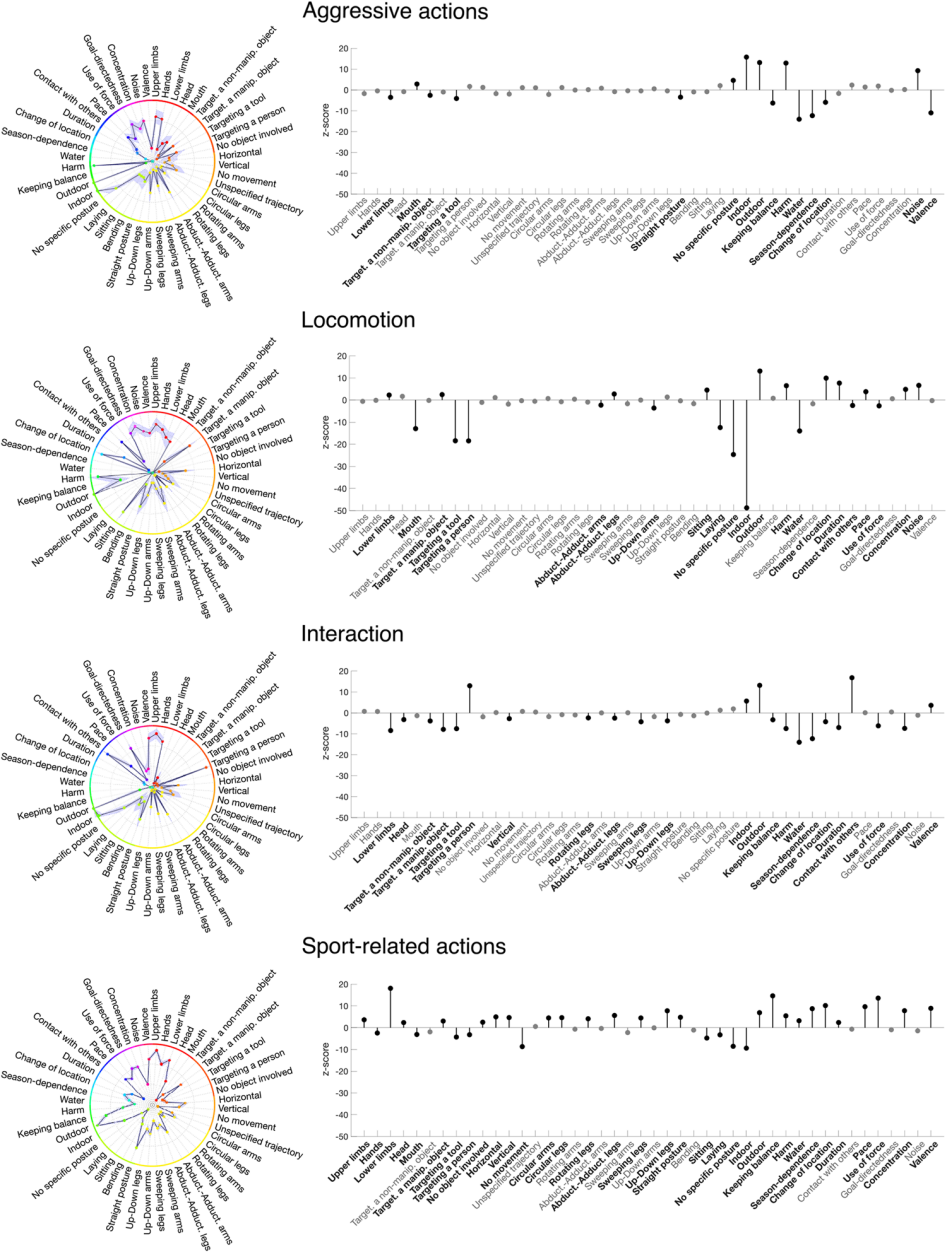
Visualization of feature vectors for selected action categories. *Left column:* Feature-based radar charts**.** *Colors* indicate features belonging to the same theme (same color code as Table 1). The length of each spike is proportional to the mean rating of the corresponding feature. *Shaded area* indicates 95% confidence interval computed across feature-based ratings of actions within a category. *Right column:* Quantification of the difference of the mean rating for features of a given category (depicted in the left column) with the mean ratings of the remaining categories, presented as z-scores. Z-scores above zero indicate higher feature ratings for a given category compared to the remaining categories, whereas z-scores below zero indicate lower feature ratings for that category compared to the remaining categories. Features that differentiate the category from the remaining categories (*p* < 0.05, FDR corrected) are marked in *black* and their corresponding names are highlighted in *bold*

The aim of this analysis was to identify features that are most important to describe action categories and to distinguish between them. First, separately for each of the categories identified in Experiment 1, we collapsed the ratings across actions within a given category. Subsequently, we used radar charts together with 95% confidence intervals (across feature ratings of actions within a category) to visualize the mean action ratings within each action category (Fig. 3, left panel, and Fig. S10).

Following Binder et al. (2016), to depict quantitative differences between each action category and the remaining categories, we computed the difference between the rating for each feature of a given category (same as depicted in Fig. 3, left panel, and Fig. S10) and the mean rating of that feature for all remaining categories (see Fig. 3, right panel and Fig. S12). The difference is presented as z-scores and the significance threshold was set at *p* < 0.05, corrected for multiple comparisons using false discovery rate (FDR) estimation (Benjamini & Hochberg, 1995).

##### Feature-based representations of single actions

To depict feature-based ratings of single actions, we computed the mean ratings of each feature across participants and mapped them on individual radar charts (see Fig. S11), separately for each action.

##### Correlation of category- and feature-based models

To determine how features contribute to explaining the category-based structure revealed by Experiment 1, we compared the results from the multi-arrangement task (Experiment 1) with the feature-based ratings (Experiment 3), using 52 different feature models: two multi-feature models (weighted and unweighted), 44 single-feature models and six theme models. As a first step, we transformed the category-based model and the feature models into RDMs (see Sections *Experiment *1*, Data analysis* and *Experiment *3*, Data analysis, Creating feature RDMs*, for details). Next, we computed the correlations between the category-based RDM and the feature RDMs using the RSA toolbox (Nili et al., 2014) and MATLAB scripts available from Storrs et al. (2020, 2021): within each cross-validation fold, together with feature weights (see Section *Experiment *3*, Data analysis, Cross-validated reweighting of features*), we calculated correlations between the feature RDMs and the category-based RDM (Kendall *τ*_*A*_) as well as the lower and upper bounds of the noise ceiling (see also Storrs et al., 2020). We ran 50 cross-validation folds, and within each fold ten randomly selected actions and five randomly selected participants were assigned as test data. At the end of the 50 cross-validation folds, the correlations, weights, and bounds of the noise ceiling were averaged. This procedure was repeated for 1000 bootstrap samples. Significance of the RDM correlations was determined by bootstrap resampling of the stimuli and controlled for multiple comparisons using FDR at 0.05.

##### Creating feature RDMs

Feature RDMs were generated by computing the Euclidean distance for each pair of actions for (1) all the unweighted features together (resulting in one unweighted multi-feature RDM), (2) all the weighted features together (resulting in one weighted multi-feature RDM (see Section *Experiment *3*, Data analysis, Cross-validated reweighting of features*), (3) each feature separately (resulting in 44 single-feature RDMs), and (4) each theme separately (resulting in six theme-based RDMs). The theme-based RDMs were computed for those themes that contained more than one feature, more precisely *Body parts*, *Object-directedness*, *Type of limb movement*, *Trajectory*, *Posture*, and *Location*. This allows investigating sets of related features together. Figure S13 shows all 52 feature RDMs (i.e., weighted and unweighted multi-feature RDMs, 44 single-feature RDMs and six theme-based RDMs).

##### Cross-validated reweighting of features

In addition to the multi-feature RDM with equal weights for each feature, we aimed to examine whether the category-based organization could be accounted for better by a weighted multi-feature RDM. Following Jozwik et al. (2016) and Storrs et al. (2021), we thus performed feature reweighting to fit the categorical action structure, while cross-validating over participants and actions. For that purpose, we used non-negative least squares fitting with the MATLAB function *lsqnonneg*. Weights were estimated for all the 44 single-feature models.

### Results

#### Multi-feature model

The multi-feature model (Fig. 2) depicts information regarding the rated contribution of features for different actions. Each row presents one action, whereas each column represents a feature. The grayscale represents the mean rating of a feature (black: “Yes/Very much”; white: “No/Not at all”). For an intuitive understanding of the model, Fig. S9 shows example actions that received the minimum and maximum rating for some exemplary features.

#### Feature-based representations of action categories

Next, we aimed to identify features that were judged as important for specific action categories. Separately for each category, we visualized feature-based ratings across actions as radar charts (see Section *Experiment *3*, Data analysis,* for details). Figure 3 (left column) shows the results for four representative action categories; Fig. S10 shows all 11 categories. As can be seen, radar charts reveal similarities as well as differences between the different categories. As an example, the features *Goal directedness*, *Upper Limbs* and *Hands* appeared to be judged as important for most categories. As can be seen in Fig. S11, showing radar charts for single actions grouped by action categories, this observation appears to be rather consistent across individual actions within each category. By contrast, other features appeared to be more distinct, such as the features *Harm* and *Noise* for the category *Aggressive actions*, *Targeting a person* and *Contact with others* for the category *Interaction*, or the feature *Keeping balance* for the category *Sport-related actions*.

Quantitative differences of feature ratings between each action category and the remaining categories are depicted in Fig. 3, right column (see Fig. S12 for all 11 categories). This comparison allows identifying the most relevant features for a given category. For example, not surprisingly, *Aggressive actions* (Fig. 3, 1st row) got significantly higher ratings on *Harm* and *Noise*, and significantly lower ratings on *Valence*, in comparison to the feature ratings obtained for the remaining categories.

For the category *Locomotion* (Fig. 3, 2nd row), this analysis revealed that the features *Change of location*, *Noise*, and *Harm* received significantly higher ratings than the remaining categories. By contrast, the feature *Indoors* was rated lower in comparison to the ratings obtained in the remaining categories. While the importance of the features *Noise* and *Harm* might be surprising at first, they are likely due to the specific actions that were part of this category (i.e., *Driving a car* and *Driving a scooter*).

For the category *Interaction* (Fig. 3, 3rd row), the analysis highlighted the importance of the features *Targeting a person* and *Contact with others*, and lower ratings for the *Use of force*, *Targeting a manipulable object*, *Lower limbs*, and *Duration*.

Finally, for the category *Sport-related actions* (Fig. 3, 4th row), pairwise comparisons revealed several movement-related features, such as *Keeping balance* and *Lower limbs*.

#### Correlation of category- and feature-based models

**Fig. 4:**
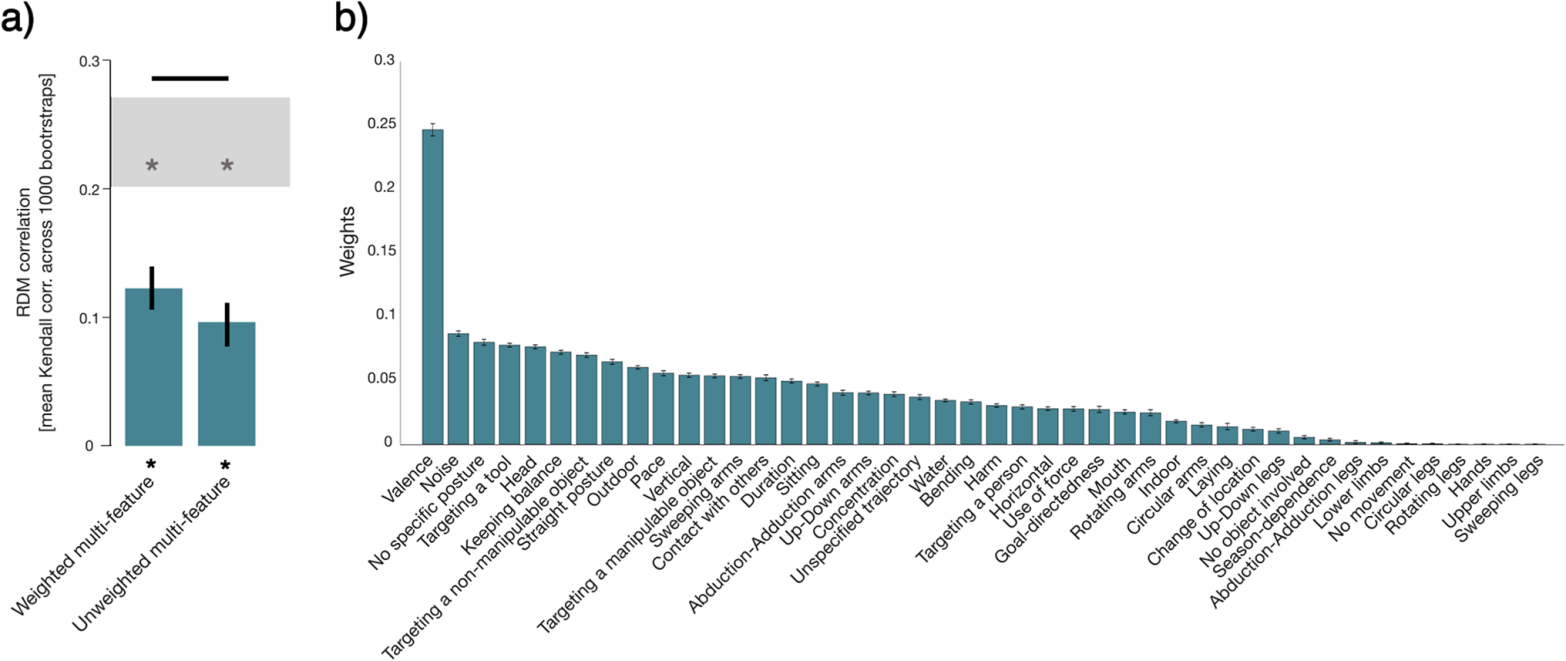
**a** Correlation between the weighted and unweighted multi-feature RDMs (obtained from Experiment 3) with the category RDM (obtained from Experiment 1). *Error bars* show the 95% confidence interval estimated by bootstrap resampling of the stimulus set. *Asterisks* at the bottom indicate that both multi-feature RDMs were significantly correlated with the category RDM (stimulus bootstrap test, *p* < 0.05, FDR corrected). The noise ceiling (*shaded area above the bars*) indicates the expected performance of the true model taking into account the noise in the data. None of the models reached the noise ceiling. The *horizontal line* above the noise ceiling indicates significant differences between the weighted and the unweighted multi-feature model (stimulus bootstrap test, *p* < 0.05, FDR corrected). Correlations between all feature RDMs and the category RDM are shown in Fig. S14. **b** Feature weights contributing to the weighted multi-feature model, obtained with non-negative least squares in the cross-validation procedure (50 cross-validation folds across participants and stimuli, averaged across 1000 bootstrapping iterations). *Error bars* indicate the 95% confidence interval of the bootstrap distribution. Only features with non-zero weights are shown

In this analysis, we aimed to determine whether and to what extent the models based on the similarity of features account for the organization based on action categories. As a first step, we computed the correlation (Kendall τ_A_) between the category RDM (obtained from Experiment 1) with the (unweighted) multi-feature RDM (obtained from Experiment 3) that consisted of all 44 features treated equally. As shown in Fig. 4a (right bar), the correlation between the unweighted multi-feature RDM and the category RDM (corr = 0.085) is significantly different from zero but does not reach the noise ceiling.

So far, we treated each feature as contributing equally to the representation of observed actions. However, it is likely that some features are shared across categories, whereas other features or combinations of features are distinct for specific categories (Tyler & Moss, 2001). Similarly to what has been proposed for object categories (Jozwik et al., 2016; Khaligh-Razavi & Kriegeskorte, 2014), action categories might differ with respect to the weights assigned to different features. To identify which features contribute the most to the categorical organization of actions revealed by Experiment 1, we thus used non-negative least-squares fitting for assigning weights to the features (see Section *Experiment *3*, Data analysis,* details) and created a weighted multi-feature model that consisted of 44 weighted features. It should be noted that feature ratings and feature weights are two separate values. In the case of feature ratings, the ratings are provided by participants and each feature is treated as contributing equally to the category-based model. However, the assumption that all features contribute equally to the category-based organization is likely to be too simplistic. The estimates of the weights of each feature, generated by non-negative linear least squares fitting, provide an insight into the importance of a given feature in explaining the category-based action representation.

As can be seen in Fig. 4a (left bar), the correlation between the weighted multi-feature RDM and the category RDM (corr = 0.121) is significantly different from zero and significantly different from the unweighted multi-feature model (calculated with stimulus bootstrap test, *p* < 0.05, FDR corrected). An overview of the significant correlations between the category model and the different feature models (unweighted multi-feature model, weighted feature model, single-feature models, and theme models) is shown in Fig. S14.

Features and the corresponding weights that formed the weighted multi-feature model are shown in Fig. 4b. The highest weight was obtained for the feature *Valence*, followed by substantially lower weights for features related to noise, posture, tool-directedness, and head.

### Discussion

The results of Experiment 3 allow narrowing down which features might contribute most to the recognition and distinction between different action categories. Additionally, a direct comparison between category- and feature-based RDMs indicated that the former can be best explained by a combination of weighted features, showing an importance of the rated valence of the action. However, part of the structure remains unexplained as evidenced by the best model not reaching the noise ceiling.

## General discussion

We performed three behavioral experiments to characterize the category- and feature-based structure underlying the organization of observed actions. Moreover, we examined the degree to which the feature-based similarity between actions accounts for the category-based organization.

In Experiment 1, we identified 11 categories, including *Communication*, *Gestures*, *Locomotion*, and *Aggressive actions* (Fig. 1). Subsets of these categories have been reported in previous studies focusing on actions depicted as pictures, videos or animations, ranging from *Hand-related actions* (Handjaras et al., 2015; Wurm et al., 2017; Wurm & Lingnau, 2015) over *Locomotion*, *Food-related actions*, *Morning routine*, and *Sport-related actions* (Abdollahi et al., 2013; Tarhan & Konkle, 2020; Tucciarelli et al., 2019) to *Interaction* (Isik et al., 2017; Papeo, 2020; Tucciarelli et al., 2019; Wurm & Caramazza, 2019). Not surprisingly, the obtained categories partly overlap with action verb categories, some of which formed the basis of stimulus selection for the current study (e.g., Vinson & Vigliocco, 2008). Although some of these category labels were similar (e.g., *Food-related actions* obtained in the current study and *Cooking* obtained from action verbs), the actions belonging to them differed: e.g., actions from the *Food-related actions* category belonged to action verb categories such as *Body-action* (e.g., *Drinking*, *Eating*, *Licking*) or *Change of state* (e.g., *Pouring*, *Stirring*). These slight differences in action categorization might depend on the stimulus material (visual vs. verbal) and the way these categories were determined (multi arrangement task of static images vs. semantic relations/ verb usage patterns). Our reason to use verbal material instead of visual stimuli to obtain features and feature ratings in Experiments 2 and 3 was to obtain features that are less dependent on the specific (visual) exemplar presented to the participant. That said, we would like to stress that action categories revealed on the basis of visual material are unlikely to be identical to the action categories revealed on the basis of verbal material (see also Tucciarelli et al., 2019; Vinson & Vigliocco, 2008; Watson & Buxbaum, 2014).

Previous studies examining dimensions underlying the organization of observed actions revealed the importance of the hand configuration and the magnitude of the arm movement for actions related to a tool (Watson & Buxbaum, 2014). For 28 daily-life actions depicted as static images, Tucciarelli et al. (2019) highlighted the type of change induced by the action and the fulfillment of basic versus higher needs. Finally, using 152 everyday action videos and an odd-one-out task combined with a data-driven approach, Dima et al. (2020) reported a number of dimensions, ranging from visual information to more social and semantic aspects of actions. A study based on large-scale text analyses revealed that actions can be described by six dimensions, including Abstraction, Food, and Spiritualizm (Thornton & Tamir, 2022). In the current study, Experiment 1 suggested that a pleasant/ non-pleasant and a tool-/ non-tool-related dimension might underlie the category-based structure. The assumption of a dimension related to pleasant/ non-pleasant actions is compatible with the results of Experiment 3 that revealed that the judged valence of an action strongly contributed to the category-based organization. It is likely that the specific dimensions revealed by different studies depend, among other things, on the type of actions included in the experiment and the methods used to reveal these dimensions.

In Experiment 2, we used a free feature-listing paradigm to identify features participants consider important for the description of actions, and for their distinction from other actions. According to the Theory of Action Identification (Vallacher & Wegner, 1985; Wegner & Vallacher, 1986), actions can be identified at different hierarchical levels that differ based on whether the feature provides information about *how* or *why* a given action is performed. According to this theory, lower identity levels are expected to rely more on movement-related information, whereas higher levels of the hierarchy are expected to be associated with a more abstract understanding of actions. Likewise, Hamilton and Grafton (2007) proposed that actions can be identified at different hierarchical levels (specifically, muscles, kinematics, goals and intentions), and that these different levels engage different brain regions. To be able to cover features from different hierarchical levels, we explicitly instructed participants to consider both concrete and abstract features (see Supplementary Materials, section S.2.1, for details on the instructions). Moreover, the obtained features cover a range of different semantic domains (e.g., Binder et al., 2016), such as sensory, motor, spatial, and temporal information.

In contrast to a selection of features purely based on previous studies, our exploratory approach for the selection of features reduces the risk of missing potentially relevant features so far not covered in the literature. The obtained set of features thus is considered to be an important extension of previous studies (e.g., Binder et al., 2016; Tarhan & Konkle, 2020; Tucciarelli et al., 2019; Vinson & Vigliocco, 2008) while serving as a basis for future experiments.

The goal of Experiment 3 was to better understand the feature-based structure of observed actions. To this aim, we collected explicit ratings for each of the 100 actions for 59 features selected on the basis of a free feature listing paradigm (Experiment 2) and features proposed in previous studies. This allowed us to compute a feature-based similarity structure of observed actions, which we used to determine (a) which features are most informative for the description of action categories and their distinction from other categories and (b) to directly compare the feature-based similarity structure with the category-based similarity structure revealed in Experiment 1. We identified a number of plausible critical features for specific categories, e.g., *Targeting a person* for the category *Interactions*; *Harm*, *Noise* and *Negative valence* for the category *Aggressive actions*; and features such as *Lower limbs*, *Keeping balance*, and *Use of force* for the category *Sport-related actions* (see Fig. 3, Fig. S10, and Fig. S12). Together, our results are in line with the view that each action category is characterized by some distinct combination of features. However, it is worth mentioning that a large proportion of the category-based structure remained unexplained. We will return to this point in the following sections.

A direct comparison of the category- and feature-based organization revealed that the weighted multi-feature model best explained the variability of the category-based structure and was significantly different from the remaining models (Fig. 4a, Fig. S14). The feature that contributed most to this organization was the valence (positive/negative) of the actions (Fig. 4b), highlighting the role of valence-related information for the categorization of actions included in the current study. In Fig. 1, we visualized the action category structure resulting from the multi-arrangement task (Experiment 1) in a two-dimensional space and referred to a pleasant/ non-pleasant dimension: Action categories associated with pleasure (e.g., *Sport-related actions*, *Hobby*, *Food-related actions*) are on the left side, whereas action categories with displeasure (e.g., *Aggressive actions*, *Household-related actions*) are on the right side. In Fig. S15, we present the same arrangement of actions in a two-dimensional space, and color-code the rated valence of each action, ranging from low (red) to high (yellow) values. This figure highlights the gradual change of valence moving from the left (positive valence) to the right (negative valence) side of the figure. Thus, the organization of actions shown in Fig. 1 and Fig. S15 might reflect whether the participants associated the actions with pleasure/ positive valence or with displeasure/ negative valence. These results are in line with previous studies examining the relationship between the processing of emotions and actions (e.g., Kroczek et al., 2021; Poyo Solanas et al., 2020). The importance of valence has also been proposed by Tamir and Thornton (2018) who suggested that valence is involved in the process of prediction other person’s actions. As suggested by the authors, understanding another person’s action relies upon understanding another person’s mental states and traits. Persons with traits of a high valence value (e.g., agreeable) most likely exhibit positive mental states (e.g., content) that lead to performing positive actions (e.g., cooperation). The authors explored dimensionalities of mental states and traits revealing the importance of valence, but they did not explore the corresponding organization of actions. In our work, we aimed to fill this gap. Based on the results from Tamir and Thornton (2018), it seems plausible that valence serves as one of the crucial dimensions underling the organization of observed action.

As mentioned above, whereas the weighted multi-feature RDM showed the highest similarity with the category RDM, a substantial part of this structure remained unexplained. In other words, even the combination of intermediate- (e.g., *Body parts*, *Type of limb movement*) and high-level features (e.g., *Valence*, *Goal-directedness*) cannot fully account for the categorical model of actions. This suggests that there must be additional organizing principles underlying the cognitive architecture of actions (see also Vinson & Vigliocco, 2008). Likely candidates that may contribute towards the organization of actions that were not specifically examined in the current study is the context or scene in which an action typically takes place (see also Tucciarelli et al., 2019; Wurm et al., 2012; Wurm & Schubotz, 2012), as well as specific objects or tools used for a given action (Bach et al., 2014; Wurm et al., 2012). We expect that more fine-grained ratings for specific scenes (e.g., kitchen, office) and objects (e.g., knife, pen), which were beyond the scope of the current study, will allow a more accurate representation of the category-based action structure.

### Future directions

Characterizing and establishing a set of action categories and features obtained from a wide range of actions, as well as better understanding of how features contribute to category-based action representation, might be useful for future behavioral and neuroimaging studies. Given the data-driven nature of Experiment 1, the individual actions that formed the categories revealed by the multi-arrangement task varied between 2 and 30, making it hard to draw firm conclusions regarding a final set of critical features for some of the categories (e.g., *Locomotion*). To be able to examine these categories in more detail, future studies should choose a more balanced number of actions per category. With these limitations in mind, we believe that our results will serve as a useful basis to stimulate future studies. As an example, future behavioral and neuroimaging studies might address how human participants process and respond to different action categories, how these capabilities develop, and under which circumstances they may become impaired. Moreover, our results might be useful in the field of computer vision and human–robot interactions. As mentioned by Wardle and Baker (2020), object recognition relies on different types of information, such as object’s appearance, task context, function of the object, and its possible interactions with other objects. The more we understand how the human brain tackles the problem of object recognition, the more accurate and human-like artificial models can be built. The same can be applied to actions: a better understanding of the way humans represent and categorize actions may lead to the creation of more accurate and efficient computational models of action recognition with higher resemblance to human’s understanding of the world. In the future, such models could be applied in automated action recognition that has become a growing demand in the industry, including autonomous vehicles and driver-assistance systems, smart surveillance, and health care systems (Serpush & Rezaei, 2021).

### Conclusions

We identified a set of action categories and showed that each of them is represented by a unique combination of action features. The reported action features can be grouped into more general themes, such as *Body parts* and *Posture*, partly overlapping with features already reported in the existing literature. Whereas the weighted multi-feature model performed best among all the examined models in explaining the category-based structure, a significant proportion of variability remained unexplained, suggesting that there are additional sources of information that contribute to the categorization of observed actions, beyond the features examined in the current study. Together, our results provide important insights into the cognitive architecture underlying our ability to distinguish between different actions and serve as an extension of the existing literature (e.g., Tucciarelli et al., 2019; Vigliocco et al., 2004; Vinson & Vigliocco, 2008). Additionally, the obtained features might be applied in computational science and help improving neural network models that could lead to more accurate computer-based action recognition in the future.

## Supplementary Information

ESM 1 (DOCX 34.8 MB)

## Data Availability

The datasets generated and analyzed during the current study are available at https://osf.io/73v58/.
