# Supplementary material for: The cognitive structure underlying the organization of observed actions
Source: Behav Res Methods. 2022 Jul 5;55(4):1890–906. doi: 10.3758/s13428-022-01894-5 (PMC10250259; doi:10.3758/s13428-022-01894-5)
Supplement: Supplementary file 1 — (DOCX 34.8 MB) [file 13428_2022_1894_MOESM1_ESM.docx]

Supplementary Materials

TABLE OF CONTENTS

S.1 Experiment 1

S.1.1 Selection of action words

Table S1. Actions used in the experiments

S.1.2 Stimulus selection

S.1.2.1 Participants

S.1.2.2 Methods

Fig S1. Stimuli used for Experiment 1

S.1.3 Procedure

Fig S2. Exemplary trial of the multi-arrangement experiment

S.1.4 Results

Fig S3. Results from the multi-arrangement task

Fig S4. Silhouette index

S.1.5 Category naming

S.1.5.1 Participants

S.1.5.2 Instruction

S.1.5.3 Apparatus

S.1.5.4 Procedure

S.1.5.5 Data analysis

Fig S5. Word cloud forms of category labels

Table S2. Labels obtained in the Category naming experiment

Table S3. List of categories and corresponding actions obtained in Experiment 1

S.2 Experiment 2

S.2.1 Instructions

S.2.2 Procedure

Fig S6. Generation of action themes and selection of key features

S.3 Experiment 3

S.3.1 Instructions

S.3.2 Data analysis

S.3.2.1 Feature redundancy removal

Table S4. List of merged binary features

Fig S7. Feature redundancy removal

S.3.2.2 Multicollinearity/Variance Inflation Factor

Table S5. Variance Inflation Factor

Fig S8. Between-feature correlations

S.3.3 Results

S.3.3.1 Multi-feature model

Fig S9. Actions that received minimum and maximum ratings for exemplary features

S.3.3.2 Feature-based representations of all 13 categories

Fig S10. Feature-based representations of all 11 action categories

S.3.3.3 Feature-based representations of all 100 actions

Fig S11. Feature-based representations of individual actions grouped by action categories

S.3.3.4 Quantitative differences between action categories

Fig S12. Quantitative differences for ratings of features of individual action categories in comparison to mean ratings obtained for the remaining categories

S.3.3.5 Feature RDMs

Fig S13 Feature RDMs used to correlate with the category model

S.3.3.6 Correlation between category-and feature-based representations

Fig S14. Correlation between category RDM and different feature RDMs

Table S6. Correlations between the category RDM and feature RDMs

S.3.3.7 Valence-based representation of actions

Fig S15. Valence-based representation of actions

S.1 Experiment 1

S.1.1 Selection of action words

Actions were chosen from a study by Vinson & Vigliocco (2008). As a first step, we discarded verbs that are difficult to depict as static images (e.g., animal sounds such as “oink”, “chirp”), with the aim to arrive at a final set of 100 actions we considered suitable for our experiments. Subsequently, we adjusted several action words towards more common actions. As an example, we chose “feeding” instead of “feeding a horse”, and “hugging” instead of “tree hugging”. Additionally, we tried to avoid keeping actions with very similar meaning in the dataset, e.g., “playing tennis” and “hitting a tennis ball”. Thus, three other actions (i.e. “hitting a tennis ball”, “playing piano”, and “fist bumping”) were removed and replaced by the actions “reading”, “riding on a bike”, and “writing on a board” from the “Stanford 40 Actions” dataset (Yao et al., 2011).

Table S1. Actions used in the experiments

1. applauding
2. arguing
3. blowing bubbles
4. breaking
5. brushing hair
6. brushing teeth
7. building a sandcastle
8. calling (phone)
9. carrying buckets
10. chopping vegetables
11. cleaning the floor
12. climbing
13. constructing
14. cooking
15. cutting trees
16. cutting with knife
17. dancing
18. digging
19. dragging
20. drawing
21. drinking
22. driving a car
23. driving a scooter
24. drumming
25. eating
26. feeding
27. fishing
28. fixing a bike
29. gardening
30. goal keeping
31. grocery shopping
32. hammering
33. hand shaking
34. handstand
35. having a shower
36. high fiving
37. hiking
38. holding hands
39. holding umbrella
40. hula hoop
41. hoovering
42. hopping
43. hugging
44. juggling
45. jumping
46. kayaking
47. kicking a football
48. knitting
49. knocking on a door
50. leaning on a hand
51. licking ice cream
52. lifting weights
53. listening to music
54. looking through microscope
55. making a bed
56. painting
57. paying someone
58. playing basketball
59. playing golf
60. playing guitar
61. playing tennis
62. pointing
63. pouring liquid
64. public speaking
65. pulling (tug of war)
66. punching
67. pushing a trolley
68. raking leaves
69. reading
70. riding a bike
71. rowing a boat
72. running
73. shooting an arrow
74. sitting
75. skateboarding
76. skiing
77. sleeping
78. sliding (water slide)
79. smoking
80. stirring
81. stroking a dog
82. surfing
83. swimming
84. swinging
85. switching on the light
86. taking a photo
87. tearing
88. texting
89. throwing a Frisbee
90. thumbs up
91. using a computer
92. walking a dog
93. washing a car
94. washing dishes
95. washing hands
96. watching TV
97. waving hand
98. writing on a board
99. writing
100. yawning

S.1.2 Stimulus selection

S.1.2.1 Participants

Nineteen healthy participants took part in the study (15 females; mean age = 22 years, age range = 18-26 years). Experimental procedures were approved by the ethics committee at the University of Regensburg.

S.1.2.2 Methods

We aimed for a final set of 100 action images depicting the actions listed in Table S1. First, we selected 160 images from Shutterstock ([www.shutterstock.com](http://www.shutterstock.com)). We chose images according to the following criteria: (1) the depicted action is the main aspect of the image, (2) the action is depicted in front of a natural (rather than a uniform) background, (3) the body of the person performing the action is fully visible, (4) there is only one person on the image (unless the action is directed at another person), and (5) the image is taken in landscape (rather than portrait) orientation. For three of the actions (*tearing*, *switching on the light* and *breaking*), we could not find suitable pictures showing the full body and thus chose images showing the upper body/arms only. To ensure that the actions were recognized as the actions we had in mind during stimulus selection, we carried out an online survey ([https://www.soscisurvey.de/](https://www.soscisurvey.de/)) with the initial set of 160 images. For actions that might be more difficult to depict as an image (e.g., arguing), we chose more than one exemplar (on average, 1.6 images per action). Participants were presented with a set of action pictures on a screen, one after the other, and were asked to type the name of the depicted action on the keyboard. The final set of stimuli was chosen by selecting, out of the set of used pictures, those for which the “correct” action label was mentioned most frequently. Based on the naming agreement, we chose a set of 100 images (see Fig S1) for the multi-arrangement task (Experiment 1).

**Fig S1. Stimuli used for Experiment 1.** Actions are sorted alphabetically (from left to right, row by row). For corresponding labels, see Table S1.

S.1.3 Procedure

| **a** | **b** |
| --- | --- |
|  |  |

**Fig S2.** **Exemplary trial of the multi-arrangement experiment (Kriegeskorte & Mur, 2012)**. Participants were asked to arrange the images by mouse drag-and-drop such that the physical distance between the images on the screen reflects the perceived similarity in terms of the meaning of the actions. In the first trial, all the 100 actions appeared on the screen around the arena. After arranging all the images within the arena, the next trial started. With each consecutive trial, a subset of images was presented, depending on pairwise dissimilarity evidence between the images (see Section Experiment 1, Procedure, for details). (**a)** Example trial with a subset of three action images (size of the images enlarged for ease of visualization). (**b)** Example arrangement at the end of a single trial.

S.1.4 Results

**Fig S3. Results from the multi-arrangement experiment.** Blue indicates low dissimilarity (high similarity), whereas yellow indicates high dissimilarity (low similarity). Labels on the x-axis are identical to labels on the y-axis.

**Fig S4.** Mean silhouette index (*si*) as a function of the number of clusters obtained from hierarchical clustering in a range from 3 to 50. For each number of clusters, the mean silhouette index was computed by averaging across 100 iterations. The red line shows the index for the number of clusters chosen in the study (*si* = 0.23).

S.1.5 Category naming

S.1.5.1 Participants

Twenty-six participants took part in the study (21 females; mean age = 26 years, age range = 18–51 years). Experimental procedures were approved by the ethics committee at the University of Regensburg.

S.1.5.2 Instruction

Participants received the following written instruction in German (for convenience, we provide the English translation):

*In this study we will ask you to find labels for sets of words. Please think about what the words have in common and try to come up with a “heading” that subsumes all of the words equally. You can give us multiple examples. Please write the label that fits best at first. There is no time limit so you can take as much time as you want. Below, you can find two examples.*

*1. Example*

- *to brake*
- *to accelerate*
- *to blink*
- *to turn*

*Possible examples for category labels could be: driving a car, riding a motorcycle, etc.*

*2. Example*

- *Karate*
- *to kick someone*
- *to hit with a fist*
- *to shout*
- *to use a weapon*
- *to break a window*

*Possible examples for category labels could be: fighting, destroying, etc.*

S.1.5.3 Apparatus

The study was conducted using an online survey ([https://www.soscisurvey.de/](https://www.soscisurvey.de/)).

S.1.5.4 Procedure

We only asked participants to provide names for clusters that contained at least two different actions. This was the case for 11 out of 12 clusters. Separately for each of the 11 clusters, participants were provided with a list of all action words (in German) belonging to a given cluster. The order of the words was randomized for each participant. Participants were instructed to provide a label that could best describe a given cluster by considering what the words had in common (see Section S.*1.5.2* for the exact wording of the instruction). Participants were allowed to provide more than one label per category.

S.1.5.5 Data analysis

In total, participants produced 493 labels (44.82 per category). To choose labels best describing each action category, we took into account the frequency of provided labels. In Fig S5 we visualized the collected category labels using word clouds, where more frequently provided labels are shown with a bigger font (in order to keep the original labels, we provide them in German). Table S2 contains all the labels provided by the participants (in German), together with the frequency of mentions. Taking into account the most frequently mentioned labels for each cluster, we selected subsequent category labels: *Aggressive actions*, *Communication*, *Food-related actions*, *Gestures*, *Hand-related actions*, *Hobby*, *Household-related actions*, *Interaction*, *Locomotion*, *Morning routine*, and *Sport-related actions*. The final list of action categories and the corresponding actions is provided in Table S3.

**Fig S5. Word cloud forms of category labels** obtained from the Category naming experiment (carried out in German). Font size is proportional to the frequency of the labels. Above each word cloud, the English translation of the label chosen for the action category is shown. For a detailed list of labels and their frequency see Table S2.

Table S2. Labels obtained in the Category naming experiment. Numbers on the left indicate the frequency of each label. Labels are organized by frequencies; labels with similar frequencies are sorted alphabetically. Labels above each list are the final action category names.

| Aggressive actions | Communication | Food-related actions |
| --- | --- | --- |
| \| 9 \| Aggression \| \| --- \| --- \| \| 9 \| Wut \| \| 7 \| Gewalt \| \| 5 \| Konflikt \| \| 4 \| Zerstörung \| \| 3 \| Aggressivität \| \| 2 \| Ärger \| \| 2 \| Zerstören \| \| 1 \| Aggressionsbewältigung \| \| 1 \| Aggressive Handlungen \| \| 1 \| Auseinandersetzung \| \| 1 \| Eskalation \| \| 1 \| Gefühlsausbruch \| \| 1 \| Gewalt ausüben \| \| 1 \| Kämpfen \| \| 1 \| Kaputt machen \| \| 1 \| negative Emotionen \| \| 1 \| physischen oder psychischen Druck ausüben \| \| 1 \| Probleme \| \| 1 \| sich aggressiv verhalten \| \| 1 \| Streit \| \| 1 \| Verletzen \| \| 1 \| Wutausbruch \| | \| 11 \| Kommunikation \| \| --- \| --- \| \| 4 \| Kommunizieren \| \| 4 \| Sprache \| \| 2 \| Mediennutzung \| \| 1 \| Alltag \| \| 1 \| Alltagsaufgaben \| \| 1 \| Arbeit \| \| 1 \| Arbeiten/ Schule \| \| 1 \| Beruf \| \| 1 \| Büro \| \| 1 \| Ideen austauschen \| \| 1 \| Informationen austauschen \| \| 1 \| Informationen generieren \| \| 1 \| Interaktion mit einer anderen Person \| \| 1 \| Kommunikationsmedien nutzen \| \| 1 \| Lehrer:in \| \| 1 \| Lesen & Schreiben \| \| 1 \| Medien benutzen \| \| 1 \| mitteilen \| \| 1 \| Office \| \| 1 \| Schreibskills \| \| 1 \| Sprachnutzung \| \| 1 \| Wissen austauschen \| \| 1 \| Wörter \| | \| 4 \| Ernährung \| \| --- \| --- \| \| 4 \| Kochen \| \| 4 \| Küche \| \| 4 \| Nahrung \| \| 3 \| Lebensmittel \| \| 3 \| Haushalt \| \| 2 \| Alltag \| \| 2 \| Essen \| \| 1 \| Essen zubereiten \| \| 1 \| Essensbezogene Handlungen \| \| 1 \| Essensvorbereitungen \| \| 1 \| Essenszubereitung \| \| 1 \| Hausarbeit \| \| 1 \| Hausarbeiten erledigen \| \| 1 \| Hunger \| \| 1 \| Küchenarbeit \| \| 1 \| Küchenarbeit verrichten / In der Küche arbeiten \| \| 1 \| Küchentätigkeiten \| \| 1 \| Leben \| \| 1 \| Lebensmittel zubereiten \| \| 1 \| Mahlzeit \| \| 1 \| Nahrungsaufnahme \| \| 1 \| Nahrungsmittelverwendung \| \| 1 \| Nahrungszubereitung und -verarbeitung \| \| 1 \| Versorgung \| \| 1 \| Zuhause \| |
| Gestures | Hand-related actions | Hobby |
| \| 4 \| Gesten \| \| --- \| --- \| \| 3 \| Kommunizieren \| \| 2 \| Handbewegung \| \| 2 \| Hände \| \| 2 \| Mitteilen \| \| 2 \| Tätigkeiten \| \| 1 \| Aktivitäten mit der Hand \| \| 1 \| Arbeit \| \| 1 \| Forschungsvortrag \| \| 1 \| Gestik \| \| 1 \| Hände bewegen / benutzen \| \| 1 \| Handmotoriken \| \| 1 \| Handnutzung \| \| 1 \| Interaktion \| \| 1 \| jemanden begrüßen \| \| 1 \| jemanden schätzen \| \| 1 \| Kommunikation \| \| 1 \| Körperliche Tätigkeiten \| \| 1 \| Präsentation \| \| 1 \| Rede halten \| \| 1 \| Referat \| \| 1 \| Schule \| \| 1 \| Sprechen \| \| 1 \| Studieren \| \| 1 \| Tagung \| \| 1 \| Uni \| \| 1 \| USA Wahlen \| \| 1 \| Versammlung \| \| 1 \| Vorlesung \| \| 1 \| Vortrag \| \| 1 \| vortragen \| \| 1 \| Wissenschaftlicher Vortrag \| \| 1 \| Wissenschaftlicher Vortrag/Ausflug \| | \| 2 \| draußen sein \| \| --- \| --- \| \| 2 \| Hände \| \| 1 \| Aktionen, die mit der Hand ausgeführt werden \| \| 1 \| Aktive Darbietung \| \| 1 \| Aktivitäten mit Händen \| \| 1 \| Ankommen \| \| 1 \| Anstand \| \| 1 \| Besuch \| \| 1 \| Fremdenführer \| \| 1 \| Geburtstagsfeier \| \| 1 \| Gegenstände benutzen \| \| 1 \| Handbewegungen im Alltag \| \| 1 \| Hände bewegen / benutzen \| \| 1 \| Handeln \| \| 1 \| händisch \| \| 1 \| Handlungen mit einer Person \| \| 1 \| Handmotorik \| \| 1 \| Handnutzung \| \| 1 \| Haptik \| \| 1 \| Hausbesuch \| \| 1 \| Interaktion mit Gegenständen \| \| 1 \| Outdoor Aktivitäten \| \| 1 \| Tätigkeiten \| \| 1 \| Tätigkeiten mit einer Hand \| \| 1 \| Vertreter \| \| 1 \| Vertreterbesuch \| \| 1 \| zielgerichtete Handlungen \| | \| 16 \| Hobby \| \| --- \| --- \| \| 8 \| Freizeit \| \| 8 \| Freizeitaktivitäten \| \| 2 \| Freizeitgestaltung \| \| 2 \| Kreativität \| \| 2 \| Spaß \| \| 1 \| Alltag \| \| 1 \| Erholung \| \| 1 \| Freizeitaktivitäten ausführen \| \| 1 \| Freizeitbeschäftigungen \| \| 1 \| kreativ \| \| 1 \| Unterhalten \| \| 1 \| Zurückzug \| |
| Household-related actions | Interaction | Locomotion |
| \| 6 \| Haushalt \| \| --- \| --- \| \| 3 \| Hausarbeit \| \| 3 \| Heimwerken \| \| 2 \| Handwerk \| \| 2 \| Instandhaltung \| \| 2 \| Ordnung \| \| 2 \| Tätigkeiten am Haus \| \| 1 \| Alltagstätigkeiten \| \| 1 \| Arbeit \| \| 1 \| außerberufliche Tätigkeiten \| \| 1 \| Bauernhof \| \| 1 \| Besitz pflegen \| \| 1 \| den Haushalt machen \| \| 1 \| Handwerken \| \| 1 \| Haus und Garten \| \| 1 \| Haus und Gartenarbeit \| \| 1 \| Haus- & Gartenarbeit verrichten \| \| 1 \| Hausarbeiten \| \| 1 \| Haushaltsaktivitäten \| \| 1 \| Haushaltsarbeit \| \| 1 \| Haushaltsarbeiten \| \| 1 \| Haushaltstätigkeiten \| \| 1 \| Heimwerkeln \| \| 1 \| Hobbys und Aktivitäten mit Händen \| \| 1 \| Körperliche Aktivität \| \| 1 \| Körperliche Arbeit am Haus und Grundstück \| \| 1 \| Körperliche Tätigkeiten \| \| 1 \| Kraft ausüben \| \| 1 \| Ordnung schaffen \| \| 1 \| Putzen \| \| 1 \| Routinearbeiten \| \| 1 \| Zuhause \| | \| 4 \| Interaktion \| \| --- \| --- \| \| 3 \| Kontakt \| \| 2 \| Geste \| \| 2 \| Interagieren \| \| 2 \| Zwischenmenschliche Aktionen \| \| 1 \| Austausch \| \| 1 \| berühren \| \| 1 \| Berührung \| \| 1 \| Beziehung \| \| 1 \| Date \| \| 1 \| Freundschaft \| \| 1 \| Handaktionen \| \| 1 \| Handbewegungen \| \| 1 \| händische Tätigkeiten \| \| 1 \| Handkontakt \| \| 1 \| Interaktion mit Menschen \| \| 1 \| Interaktion zwischen menschen \| \| 1 \| jemanden treffen \| \| 1 \| Kontakt mit Menschen \| \| 1 \| Körperkontakt \| \| 1 \| sich berühren \| \| 1 \| sich mit anderen Personen austauschen \| \| 1 \| soziale Interaktion \| \| 1 \| Tätigkeiten mit den Händen und Armen \| \| 1 \| Verabschiedung \| \| 1 \| Verbundenheit \| \| 1 \| Wertschätzung zeigen \| \| 1 \| Zusammenarbeiten \| \| 1 \| Zusammenhalt \| \| 1 \| Zwischenmenschlicher Austausch \| | \| 9 \| Fortbewegung \| \| --- \| --- \| \| 5 \| Fahren \| \| 3 \| Transportmittel \| \| 3 \| Verkehrsmittel \| \| 2 \| Fahrzeug fahren \| \| 2 \| Fortbewegen \| \| 2 \| Lenken \| \| 2 \| Mobilität \| \| 2 \| sich fortbewegen \| \| 2 \| Straßenverkehr \| \| 2 \| unterwegs sein \| \| 1 \| am Straßenverkehr teilnehmen \| \| 1 \| Fahrzeug benutzen \| \| 1 \| Fortbewegungsmöglichkeiten \| \| 1 \| Führerschein \| \| 1 \| Lokomotion \| \| 1 \| Maschinelle Fortbewegung \| \| 1 \| Motorisierte Transportmittel \| \| 1 \| Personentransport \| \| 1 \| Reise \| \| 1 \| Straße benutzen \| \| 1 \| Transport \| \| 1 \| Transportieren \| \| 1 \| Transportmittel benutzen \| \| 1 \| Verkehr \| |
| Morning routine | Sport-related actions |  |
| \| 8 \| Morgenroutine \| \| --- \| --- \| \| 5 \| Aufstehen \| \| 3 \| Abend \| \| 3 \| Abendroutine \| \| 1 \| Aktionen \| \| 1 \| Aktivitäten \| \| 1 \| Alltag \| \| 1 \| Alltagshandlungen ausführen \| \| 1 \| aufwachen \| \| 1 \| Bad \| \| 1 \| Badezimmer \| \| 1 \| Bettfertig machen \| \| 1 \| Fertig machen für den Tag \| \| 1 \| Handeln \| \| 1 \| Handlungen \| \| 1 \| Hygiene \| \| 1 \| Ins Bett gehen \| \| 1 \| Körperliche Bewegung \| \| 1 \| Körperliche Tätigkeiten \| \| 1 \| Morgen \| \| 1 \| Morgens \| \| 1 \| morgens aufstehen \| \| 1 \| Nachtschlafvorbereitung \| \| 1 \| Regeneration \| \| 1 \| runterfahren \| \| 1 \| Schlafen gehen \| \| 1 \| Schlafzimmer \| \| 1 \| sich fertig machen für das Schlafen \| \| 1 \| tägliche Routine \| \| 1 \| Tätigkeiten \| \| 1 \| Wach werden \| | \| 15 \| Sport \| \| --- \| --- \| \| 8 \| Hobby \| \| 4 \| Freizeit \| \| 3 \| Aktivitäten \| \| 3 \| Bewegung \| \| 2 \| Freizeitaktivitäten \| \| 2 \| Körperliche Aktivität \| \| 2 \| Urlaub \| \| 1 \| Abenteuer \| \| 1 \| Bewegen \| \| 1 \| Fitness \| \| 1 \| Freizeitaktivitäten ausführen \| \| 1 \| Körperbezogene Aktivitäten \| \| 1 \| Körpereinsatz Bewegung \| \| 1 \| Körperliche Betätigung \| \| 1 \| Leibesübungen \| \| 1 \| Outdooraktivitäten \| \| 1 \| sich betätigen \| \| 1 \| sich bewegen \| \| 1 \| Spaß \| \| 1 \| Spiel \| \| 1 \| Sport machen \| \| 1 \| Sport treiben \| \| 1 \| Sportaktivitäten \| \| 1 \| Sportarten \| \| 1 \| sportliche Aktivitäten \| \| 1 \| Sportliche Tätigkeiten \| |  |

Table S3. List of categories and corresponding actions obtained in Experiment 1.

| **Category** | **Actions** |
| --- | --- |
| **Aggressive actions** | Arguing; Breaking; Punching; Tearing |
| **Communication** | Calling; Reading; Texting; Using a computer; Writing on a board |
| **Food-related actions** | Chopping vegetables; Cooking; Cutting with knife; Drinking; Eating; Feeding; Grocery shopping; Licking ice cream; Pouring liquid; Stirring; Washing dishes |
| **Gestures** | Applauding; Hand shaking; Leaning on a hand; Looking through microscope; Public speaking; Thumbs up; Waving hand; Writing |
| **Hand-related actions** | Holding umbrella; Knocking on a door; Pointing |
| **Hobby** | Dancing; Drawing; Drumming; Knitting; Listening to music; Painting; Playing guitar; Stroking a dog; Taking a photo; Walking a dog; Watching TV |
| **Household-related actions** | Carrying buckets; Cleaning the floor; Constructing; Cutting trees; Digging; Dragging; Fixing a bike; Gardening; Hammering; Hoovering; Making a bed; Pushing a trolley; Raking leaves; Washing a car |
| **Interaction** | High-fiving; Holding hands; Hugging; Paying someone |
| **Locomotion** | Driving a car; Driving a scooter |
| **Morning routine** | Brushing hair; Brushing teeth; Sitting; Sleeping; Switching on the light; Washing hands; Yawning |
| **Sport-related actions** | Blowing bubbles; Building a sandcastle; Climbing; Fishing; Goal keeping; Handstand; Having a shower; Hiking; Hula-hoop; Hopping; Juggling; Jumping; Kayaking; Kicking a football; Lifting weights; Playing basketball; Playing golf; Playing tennis; Pulling; Riding a bike; Rowing a boat; Running; Shooting an arrow; Skateboarding; Skiing; Sliding (water sliding); Surfing; Swimming; Swinging; Throwing a Frisbee |
| **Cluster containing one action** | Smoking |

Hierarchical clustering resulted in 12 action categories (see left column). Actions belonging to a given category are provided in the right column. Categories containing at least two actions (11 categories) were used in the Category naming experiment, which was performed to generate category labels (corresponding names are provided in the left column, sorted alphabetically). One cluster that consisted of one action only (*smoking*) was discarded from further analyses (highlighted in gray, shown at the bottom of the table).

S.2 Experiment 2

S.2.1 Instructions

Participants taking part in the feature generation task received the following instructions (in German):

*During the experiment, you will see a set of 25 action words and you will be asked to write down features of each action. You should write all the features which you think are relevant to describe a given action and to distinguish this action from the others. Please consider both abstract features (such as “sociality”, “transitivity”) and more detailed, concrete features (such as “moving fingers”, “lifting up arms”). Please try to imagine a given action in many different scenes and write down features that are common.*

*You can type the features in boxes placed next to the action word. Please type as many features as possible, with a minimum of 5 per word. See the examples to get an idea of how to describe the action words in terms of their features.*

***Playing piano****: Music related, Body-sense, Finger movement, Rapid change, Being focused*

***Talking****: Sociality, Contact, Communication, Mouth movement, Eye contact*

S.2.2 Procedure

**Fig S6. Generation of action themes and selection of key features.** First, participants listed at least five features per action word (“Getting action features”), resulting in 5683 features in total. Next (“Preparing the dataset”), for each action, features with a similar meaning were merged, resulting in 4505 unique features (3243 unique features within the whole dataset). Guided by features examined in previous studies and the frequency of unique features, we selected a total of 59 features (see fourth column for examples), which we organized according to 19 broad action themes (see third column for examples).

S.3 Experiment 3

S.3.1 Instructions

Participants received the following written instructions (in German):

*In this study, you will be asked to describe different actions based on ratings in different themes. These themes include various aspects of actions, such as: involved body parts, duration of the action, its speed, etc.*

*The experiment takes approximately 45 minutes and consists of 25 actions. In the course of the experiment, you may be asked to rate the same actions more than once.*

*Below you will find an example of the action "applying make-up" with sample answers. Please take a look at this example and the explanations of the themes to better understand what each theme means.*

*Unless otherwise stated, you can select more than one option for each theme.*

S.3.2 Data analysis

S.3.2.1 Feature redundancy removal

During the feature rating, features for some of the themes (e.g., *Far awa*y, *In proximity*, *No change of location* for the theme *Change of location*; see Table S4) required binary judgments. To reduce the redundancy within the dataset, we merged features that could be expressed on a single scale. As a first step, we assigned a number to each of the features within a theme (e.g., 3, 2, 1 for the features *Far away*, *In proximity*, *No change*; see Fig S7) and subsequently transformed the ratings to one rating depictable on a scale. Fig S7 provides two example ratings. In the first example (for the action *Driving a car*), the participant indicated “Yes” for the feature *Far away*, and a “No” for the features *In proximity* and *No change of location*. This answer was transformed to the new rating “3”. In the second example (for the action *Arguing*), the participant gave the answer “No” for the feature *Far away*, and the answer “Yes” for the features *In proximity* and *No change of location*. In this case, the answer was transformed to the new rating “1.5” (i.e., the mean of the values corresponding to the two features judged with “Yes”). As a result, the number of features was reduced to 49, and nine of them could be depicted on a discrete scale. In the next step, we re-scaled ratings of all 49 features to a range of 0-1.

Table S4. List of merged binary features.

| **Original features** | **Location on the new scale** | **Merged features** |
| --- | --- | --- |
| Far away  In proximity  No change of location | 3  2  1 | Change of location |
| A day  Several hours  Half an hour to an hour  A few minutes to half an hour  A few seconds to a few minutes  Up to a few seconds | 6  5  4  3  2  1 | Duration |
| Touching another person  Contact from a distance  Indirect contact  Does not require contact | 4  3  2  1 | Contact with others |

Numbers in the middle column indicate location of each “old” feature on the scale of the new theme. New feature labels are provided in the right column.


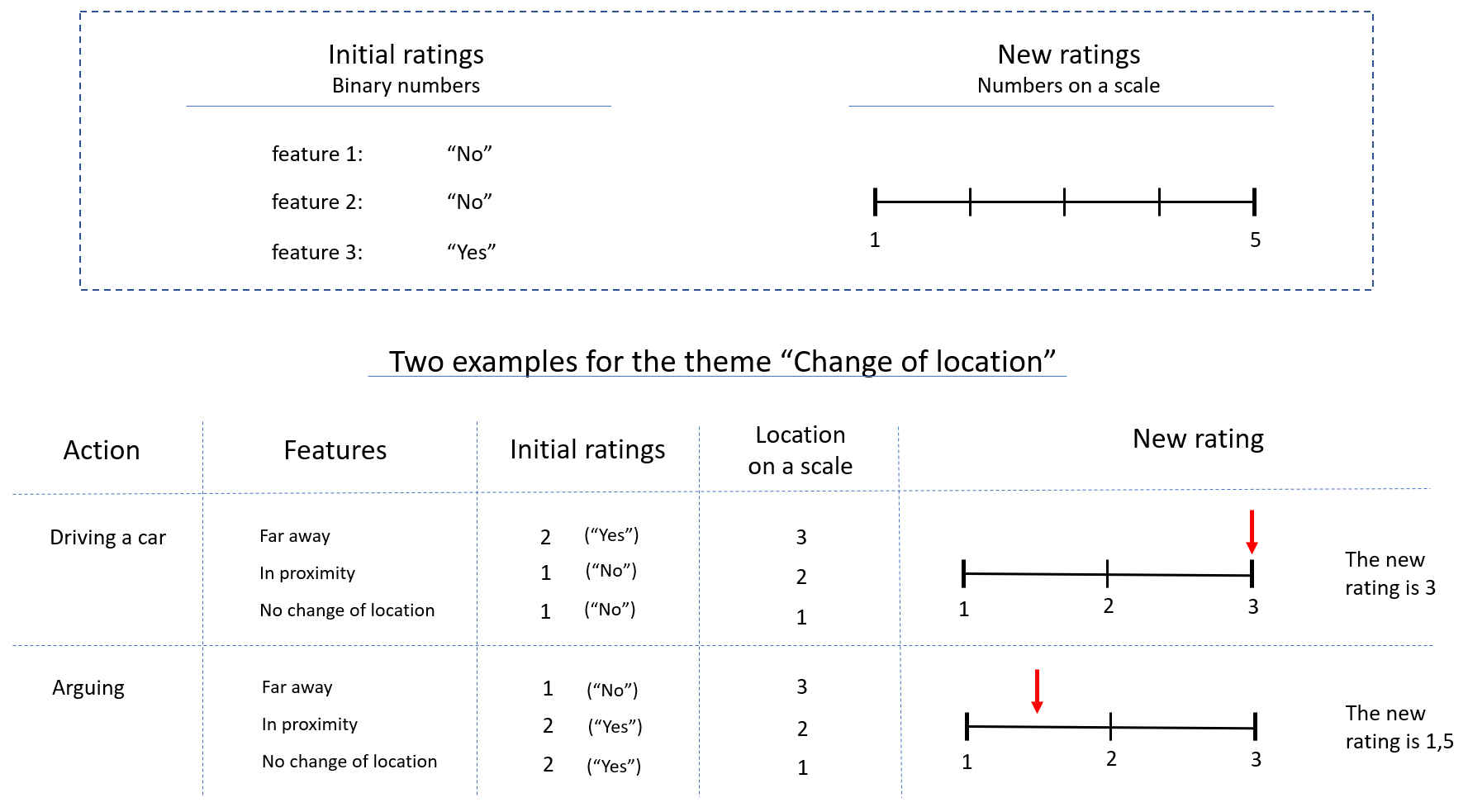
**Fig S7.** **Feature redundancy removal.** See text above for details.

S.3.2.2 Multicollinearity/Variance Inflation Factor

To estimate the amount of multicollinearity between feature ratings we computed the Variance Inflation Factor (VIF) (Table S5a) and between-feature correlations using Pearson correlation (Fig S8a). For features with a VIF > 10 and significant between-feature correlations (p < 0.05), we collapsed ratings. The ratings were collapsed at the level of individual participants. This resulted in the features *Upper limbs* (by collapsing *Arms* and *Shoulders*), *Hands* (by collapsing *Dominant hand*, *Both hands*, and *Fingers*) and *Lower limbs* (by collapsing *Legs*, *Hips*, and *Feet*), for a final set of 44 features in total. Results of the VIF for these merged features are shown in Table S5b and results for the between-feature correlations are provided in Fig S8b.

Table S5. Variance Inflation Factor. (a) Before and (b) after collapsing features.

| **a** | **b** |
| --- | --- |
| \| **Feature** \| **VIF** \| \| --- \| --- \| \| Arms  Shoulder  Dominant hand  Both hands  Fingers  Legs  Hips  Feet  Head  Mouth  Targeting a non-manip. object  Targeting a manip. object  Targeting a tool  Targeting a person  No object involved  Horizontal  Vertical  No movement  Unspecified trajectory  Circular arms  Circular legs  Rotating arms  Rotating legs  Abduction-Adduction arms  Abduction-Adduction legs  Sweeping arms  Sweeping legs  Up-Down arms  Up-Down legs  Straight posture  Bending  Sitting  Laying  No specific posture  Indoor  Outdoor  Keeping balance  Harm  Water  Season-dependence  Change of location  Duration  Contact with others  Pace  Use of force  Goal-directedness  Concentration  Noise  Valence \| 6.365  6.441  11.152  4.894  11.696  58.000  45.948  33.369  5.310  3.590  3.182  4.610  2.694  6.570  6.098  6.533  4.979  12.164  3.295  4.746  4.392  5.637  5.745  3.404  6.892  4.706  5.572  4.512  5.566  4.122  3.789  3.371  3.214  4.436  10.497  2.762  4.706  3.393  2.591  6.964  6.827  6.451  5.168  4.880  6.600  3.269  3.676  3.942  2.577 \| | \| **Feature** \| **VIF** \| \| --- \| --- \| \| Upper limbs  Hands  Lower limbs  Head  Mouth  Targeting a non-manip. object  Targeting a manip. object  Targeting a tool  Targeting a person  No object involved  Horizontal  Vertical  No movement  Unspecified trajectory  Circular arms  Circular legs  Rotating arms  Rotating legs  Abduction-Adduction arms  Abduction-Adduction legs  Sweeping arms  Sweeping legs  Up-Down arms  Up-Down legs  Straight posture  Bending  Sitting  Laying  No specific posture  Indoor  Outdoor  Keeping balance  Harm  Water  Season-dependence  Change of location  Duration  Contact with others  Pace  Use of force  Goal-directedness  Concentration  Noise  Valence \| 5.301  5.339  10.663  4.665  2.984  2.379  3.863  2.513  6.103  4.461  5.944  4.411  11.169  3.077  4.242  3.802  5.082  5.208  2.945  5.623  4.260  4.248  3.805  5.316  3.715  2.928  3.214  2.980  4.962  8.321  2.703  4.426  3.268  2.316  5.286  6.661  5.881  4.874  3.861  5.568  3.016  3.262  2.949  2.411 \| |

Different colors (i.e. yellow, green, and blue) indicate which features were merged together (column **a**), and which final features were formed based on them (column **b**).

| **a** | **** |
| --- | --- |
| **b** |  |

**Fig S8. Between-feature correlations**. Stars indicate significant correlation between features, corrected for multiple comparisons (FDR, p < 0.05). **(a)** Before averaging across highly correlated features (see Section S.3.2.2). **(b)** After averaging across highly correlated features.

S.3.3 Results

S.3.3.1 Multi-feature model

**Fig S9.** Actions that received minimum (left column) and maximum (right column) ratings for some exemplary features (*Change of location*, *Contact with others*, *Noise*, *Valence*).

S.3.3.2 Feature-based representations of all 11 categories

**Fig S10. Feature-based representations of all 11 action categories.** Different colors indicate features belonging to the same theme. The length of the spikes corresponds to the averaged rating of the corresponding feature for that category. Shaded area indicates a 95% confidence interval across actions within each category.

S.3.3.3 Feature-based representations of all 100 actions

**Fig S11. Feature-based representations of individual actions grouped by action categories**. One hundred actions are depicted as radial plots and grouped by the categories obtained from Experiment 1. Radial plots show the importance of various features (obtained from Experiment 2) ranging from 0 to 1. Features are color-coded, with features belonging to one theme indicated by the same color (same color code as Fig 2). A list of the themes and corresponding colors is provided in the legend of the exemplary radial plot on the top of the figure.

S.3.3.4 Quantitative differences between action categories

**Fig S12.** Quantitative differences for feature ratings of individual action categories in comparison to mean ratings obtained for the remaining categories, expressed as z-scores. This comparison reveals crucial features that distinguish between action categories. Significant pairwise comparisons are indicated by bold feature labels on the x axes (p < 0.05, FDR corrected).

S.3.3.5 Feature RDMs

**Fig S13.** Feature RDMs used to correlate with the category model: unweighted multi-feature RDM, weighted multi-feature RDM, 44 single-feature RDMs, and six theme RDMs. Black squares indicate high similarity between the actions whereas white squares indicate low similarity. The RDMs were obtained by computing the Euclidean distance between pairs of actions.

S.3.3.6 Correlation between category- and feature-based representations

**Fig S14.** Correlation between category RDM (resulting from Experiment 1) and different feature RDMs (see Section *Experiment 3, Data analysis, Correlation of category- and feature-based models*, for details). The figure is an extension of Fig 4a. Significant differences between the feature RDMs are indicated by horizontal lines above the bars (stimulus bootstrap test, p < 0.05, FDR corrected).

Table S6. Correlations between the category RDM (resulting from Experiment 1) and feature RDMs (resulting from Experiment 3). Feature RDMs are organized in a descending manner (same order as in Figure S14).

| **Feature RDM correlated with category RDM** | **Kendall’s τ_A_ correlation** |
| --- | --- |
| Weighted multi-feature | 0.1206 |
| Valence | 0.0926 |
| Unweighted multi-feature | 0.0850 |
| Keeping balance | 0.0701 |
| Lower limbs | 0.0671 |
| No specific posture | 0.0621 |
| Use of force | 0.0588 |
| Body parts | 0.0573 |
| Object-directedness | 0.0573 |
| Trajectory | 0.0573 |
| Type of limb movement | 0.0573 |
| Posture | 0.0573 |
| Location | 0.0573 |
| Noise | 0.0474 |
| No movement | 0.0467 |
| Harm | 0.0421 |
| Straight posture | 0.0417 |
| Concentration | 0.0386 |
| Pace | 0.0367 |
| Laying | 0.0354 |
| Targeting a manipulable object | 0.0348 |
| Targeting a person | 0.0318 |
| Head | 0.0314 |
| Upper limbs | 0.0281 |
| Change of location | 0.0279 |
| Contact with others | 0.0249 |
| Indoor | 0.0238 |
| Targeting a non-manipulable object | 0.0203 |
| Targeting a tool | 0.0198 |
| Sitting | 0.0197 |
| Up-Down legs | 0.0193 |
| Mouth | 0.0157 |
| Abduction-Adduction legs | 0.0135 |
| Horizontal | 0.0135 |
| Vertical | 0.0134 |
| No object involved | 0.0118 |
| Duration | 0.0116 |
| Outdoor | 0.0100 |
| Circular legs | 0.0086 |
| Sweeping legs | 0.0069 |
| Goal-directedness | 0.0060 |
| Bending | 0.0045 |
| Rotating legs | 0.0042 |
| Sweeping arms | 0.0021 |
| Hands | 0.0020 |
| Abduction-Adduction arms | 0.0017 |
| Season-dependence | 0.0017 |
| Circular arms | 0.0003 |
| Rotating arms | -0.0015 |
| Up-down arms | -0.0051 |
| Water | -0.0058 |
| Unspecified trajectory | -0.0091 |

S.3.3.7 Valence-based representation of actions

**Fig S15. Valence-based representation of actions.** 2-dimensional arrangement of actions obtained from the multi-arrangement experiment (Experiment 1; same representation of actions as shown in Fig 1), color-coded with respect to the valence ratings obtained from Experiment 3 (red: *negative valence*, yellow: *positive valence*). Based on the importance of the feature valence revealed by the results shown in Figure 4b, this visualization aims to better understand the organization of observed actions according to this feature.

References

Vinson, D. P., & Vigliocco, G. (2008). Semantic feature production norms for a large set of objects and events. *Behavior Research Methods*. https://doi.org/10.3758/BRM.40.1.183

Yao, B., Jiang, X., Khosla, A., Lin, A. L., Guibas, L., & Fei-Fei, L. (2011). Human action recognition by learning bases of action attributes and parts. In *Proceedings of the IEEE International Conference on Computer Vision*. https://doi.org/10.1109/ICCV.2011.6126386

Figure captions

**Fig S1. Stimuli used for Experiment 1.** Actions are sorted alphabetically (from left to right, row by row). For corresponding labels, see Table S1.

**Fig S2.** **Exemplary trial of the multi-arrangement experiment (Kriegeskorte & Mur, 2012)**. Participants were asked to arrange the images by mouse drag-and-drop such that the physical distance between the images reflects the perceived similarity in terms of the meaning of the actions. In the first trial, all the 100 actions appeared on the screen around the arena. After arranging all the images within the arena, the next trial started. With each consecutive trial, a subset of images was presented, depending on pairwise dissimilarity evidence between the images (see Section Experiment 1, Procedure, for details). (**a)** Example trial with a subset of three action images (size of the images enlarged for ease of visualization). (**b)** Example arrangement at the end of a single trial.

**Fig S3. Results from the multi-arrangement experiment.** Blue indicates low dissimilarity (high similarity), whereas yellow indicates high dissimilarity (low similarity). Labels on the x-axis are identical to labels on the y-axis.

**Fig S4.** Mean silhouette index (*si*) as a function of the number of clusters obtained from hierarchical clustering in a range from 3 to 50. For each number of clusters, the mean silhouette index was computed by averaging across 100 iterations. The red line shows the index for the number of clusters chosen in the study (*si* = 0.23).

**Fig S5. Word cloud forms of category labels** obtained from the Category naming experiment (carried out in German). Font size is proportional to the frequency of the labels. Above each word cloud, the English translation of the label chosen for the action category is shown. For a detailed list of provided labels and their frequency see Table S2.

**Fig S6. Generation of action themes and selection of key features.** First, participants listed at least five features per action word (“Getting action features”), resulting in 5683 features in total. Next (“Preparing the dataset”), for each action, features with a similar meaning were merged, resulting in 4505 unique features (3243 unique features within the whole dataset). Guided by features examined in previous studies and the frequency of features in the feature generation task, we selected a total of 59 features (see fourth column for examples), which we organized according to 19 broad action themes (see third column for examples).

**Fig S7.** **Feature redundancy removal.** See text above for details.

**Fig S8. Between-feature correlations**. Stars indicate significant correlation between features, corrected for multiple comparisons (FDR, p < 0.05). **(a)** Before averaging across highly correlated features (see Section S.3.2.2). **(b)** After averaging across highly correlated features.

**Fig S9.** Actions that received minimum (left column) and maximum (right column) ratings for some exemplary features (*Change of location*, *Contact with others*, *Noise*, *Valence*).

**Fig S10. Feature-based representations of all 11 action categories.** Different colors indicate features belonging to the same theme. The length of the spikes corresponds to the averaged rating of the corresponding feature for that category. Shaded area indicates a 95% confidence interval across actions within each category.

**Fig S11. Feature-based representations of individual actions grouped by action categories**. One hundred actions are depicted as radial plots and grouped by the categories obtained from Experiment 1. Radial plots show the importance of various features (obtained from Experiment 2) ranging from 0 to 1. Features are color-coded, with features belonging to one theme indicated by the same color (same color code as Fig 2). A list of the themes and corresponding colors is provided in the legend of the exemplary radial plot on the top of the figure.

**Fig S12.** Quantitative differences for feature ratings of individual action categories in comparison to mean ratings obtained for the remaining categories, expressed as z-scores. This comparison reveals crucial features that distinguish between categories. Significant pairwise comparisons are indicated by bold feature labels on the x axes (p < 0.05, FDR corrected).

**Fig S13.** Feature RDMs used to correlate with the category model: unweighted multi-feature RDM, weighted multi-feature RDM, 44 single-feature RDMs, and six theme RDMs. Black squares indicate high similarity between the actions whereas white squares indicate low similarity. The RDMs were obtained by computing the Euclidean distance between pairs of actions.

**Fig S14.** Correlation between category RDM (resulting from Experiment 1) and different feature RDMs (see Section *Experiment 3, Data analysis, Correlation of category- and feature-based models,* for details). The figure is an extension of Fig 4a. Significant differences between the feature RDMs are indicated by horizontal lines above the bars (stimulus bootstrap test, p < 0.05, FDR corrected).

**Fig S15. Valence-based representation of actions.** 2-dimensional arrangement of actions obtained from the multi-arrangement experiment (Experiment 1; same representation of actions as shown in Fig 1), color-coded with respect to the valence ratings obtained from Experiment 3 (red: *negative valence*, yellow: *positive valence*). Based on the importance of the feature valence revealed by the results shown in Figure 4b, this visualization aims to better understand the organization of observed actions according to this feature.
